# Supplementary material for: Mendelian randomization analysis reveals causal association of anthropometric measures on sepsis risk and mortality
Source: PLoS One. 2024 Sep 30;19(9):e0310898. doi: 10.1371/journal.pone.0310898 (PMC11441680; doi:10.1371/journal.pone.0310898)
Supplement: S1 File — (DOCX) [file pone.0310898.s003.docx]

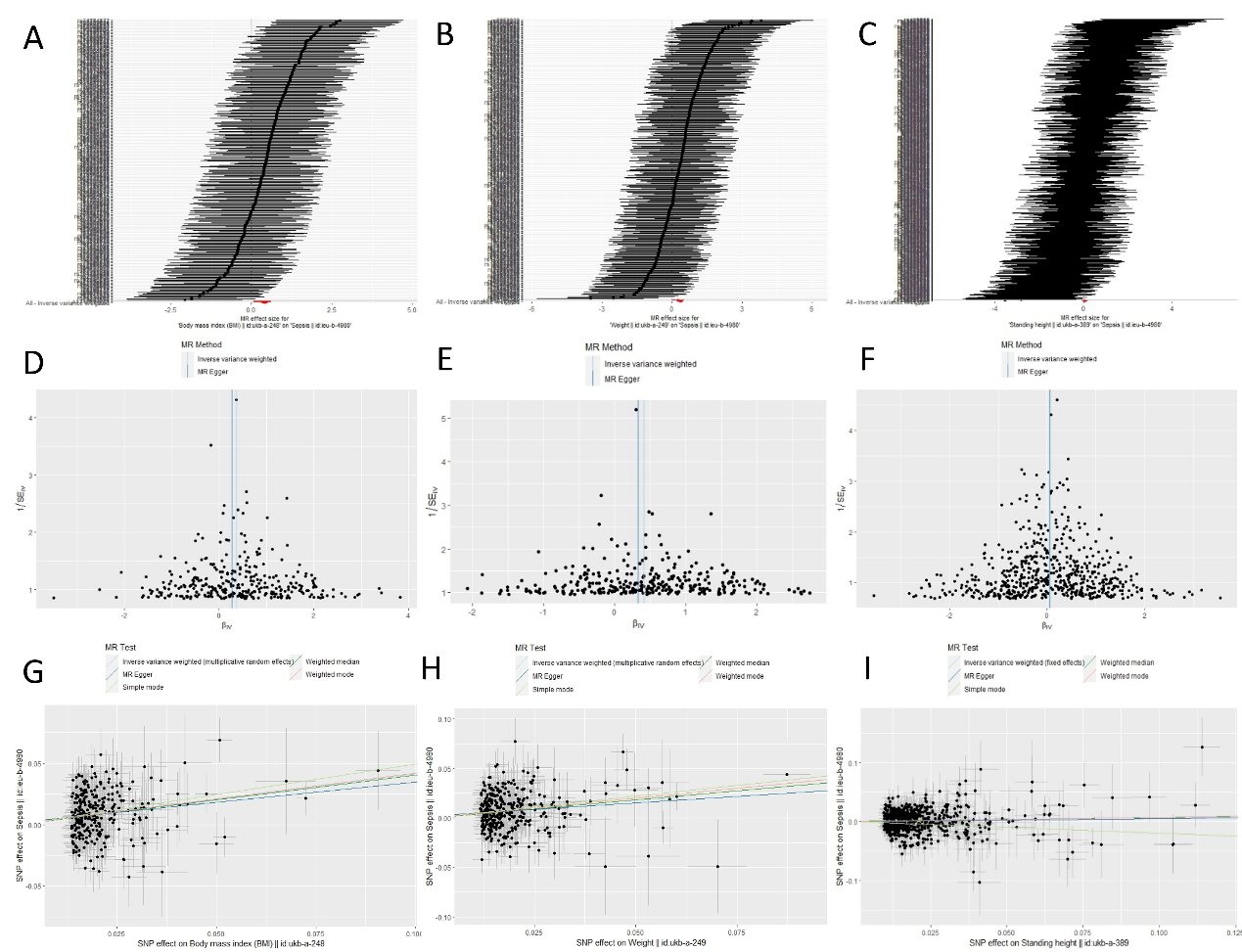


Supplementary Fig 1 The visualization for the causal effect of general anthropometric measures (BMI, weight and height) on sepsis risk. (A-C) Forest plot to visualize causal effect of each SNP on sepsis risk. (A) Causal effect of BMI on sepsis risk. (B) Causal effect of weight on sepsis risk. (C) Causal effect of height on sepsis risk. (D-F) Funnel plots to visualize overall heterogeneity of MR estimates for the effect of BMI, weight and height on sepsis risk. (D) Causal effect of BMI on sepsis risk. (E) Causal effect of weight on sepsis risk. (F) Causal effect of height on sepsis risk. (G-I) Scatter plot showing the causality of BMI, weight and height on sepsis risk. (G) Causal effect of BMI on sepsis risk. (H) Causal effect of weight on sepsis risk. (I) Causal effect of height on sepsis risk. IVW, inverse-variance weighted; MR, Mendelian randomization.


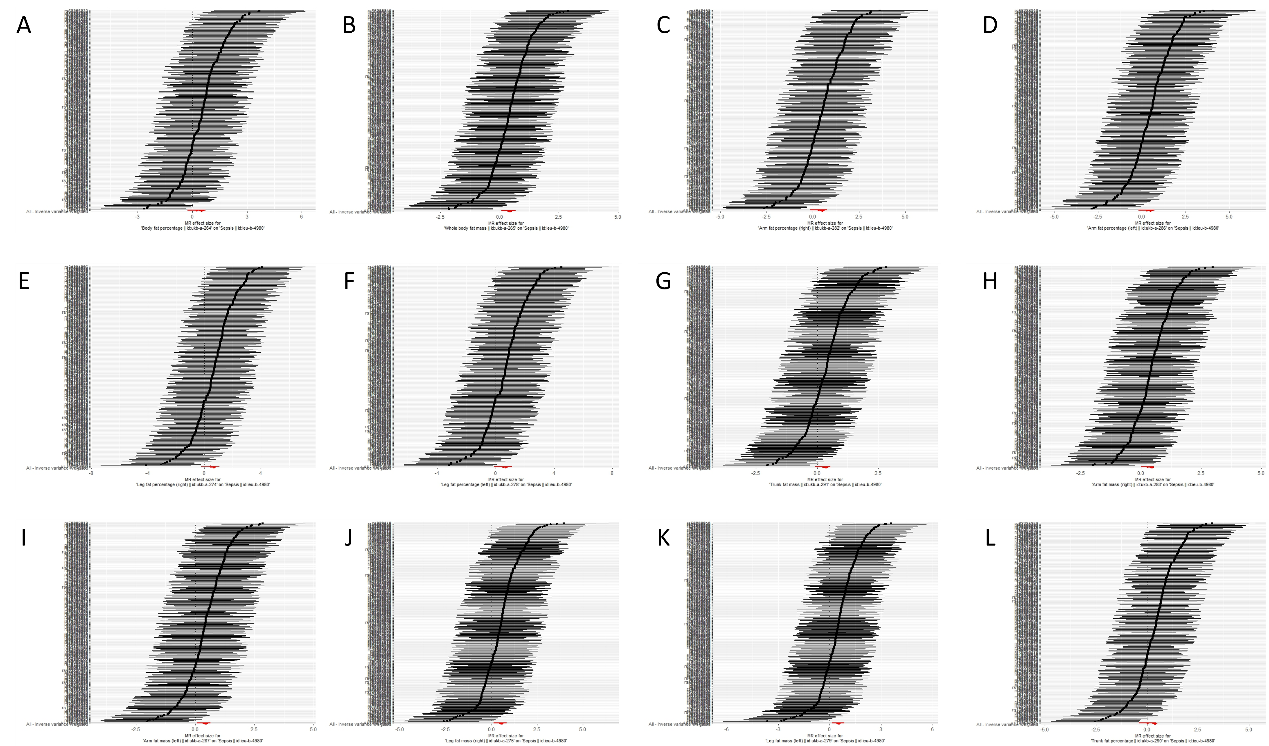


Supplementary Fig 2 Forest plot to visualize causal effect of each SNP on sepsis risk. (A) Causal effect of body fat percentage on sepsis risk. (B) Causal effect of Body fat mass on sepsis risk. (C) Causal effect of arm fat percentage (right) on sepsis risk. (D) Causal effect of arm fat percentage (left) on sepsis risk. (E) Causal effect of leg fat percentage (right) on sepsis risk. (F) Causal effect of leg fat percentage (left) on sepsis risk. (G) Causal effect of trunk fat mass on sepsis risk. (H) Causal effect of arm fat mass (right) on sepsis risk. (I) Causal effect of arm fat mass (left) on sepsis risk. (G) Causal effect of leg fat mass (right) on sepsis risk. (K) Causal effect of leg fat mass (left) on sepsis risk. (L) Causal effect of trunk fat percentage on sepsis risk. IVW, inverse-variance weighted; MR, Mendelian randomization.


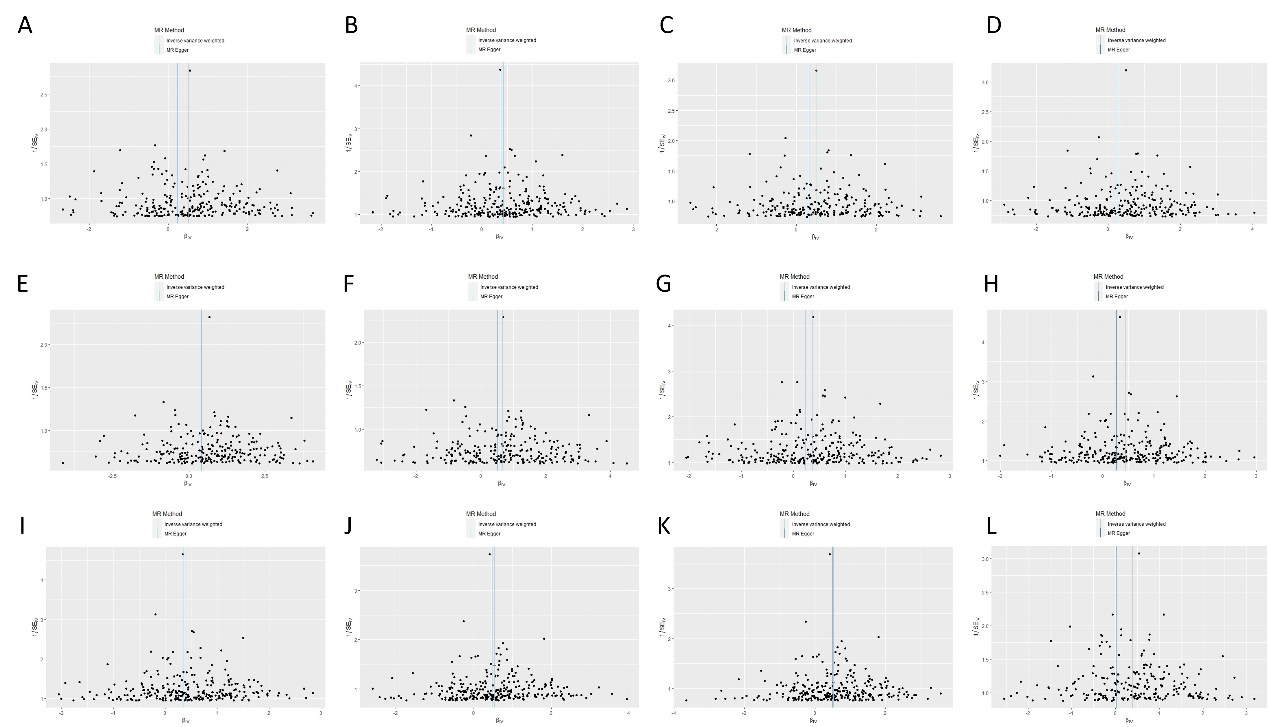


Supplementary Fig 3 Funnel plots to visualize overall heterogeneity of MR estimates for the effect of fat mass on sepsis risk. (A) Causal effect of body fat percentage on sepsis risk. (B) Causal effect of Body fat mass on sepsis risk. (C) Causal effect of arm fat percentage (right) on sepsis risk. (D) Causal effect of arm fat percentage (left) on sepsis risk. (E) Causal effect of leg fat percentage (right) on sepsis risk. (F) Causal effect of leg fat percentage (left) on sepsis risk. (G) Causal effect of trunk fat mass on sepsis risk. (H) Causal effect of arm fat mass (right) on sepsis risk. (I) Causal effect of arm fat mass (left) on sepsis risk. (G) Causal effect of leg fat mass (right) on sepsis risk. (K) Causal effect of leg fat mass (left) on sepsis risk. (L) Causal effect of trunk fat percentage on sepsis risk. IVW, inverse-variance weighted; MR, Mendelian randomization.


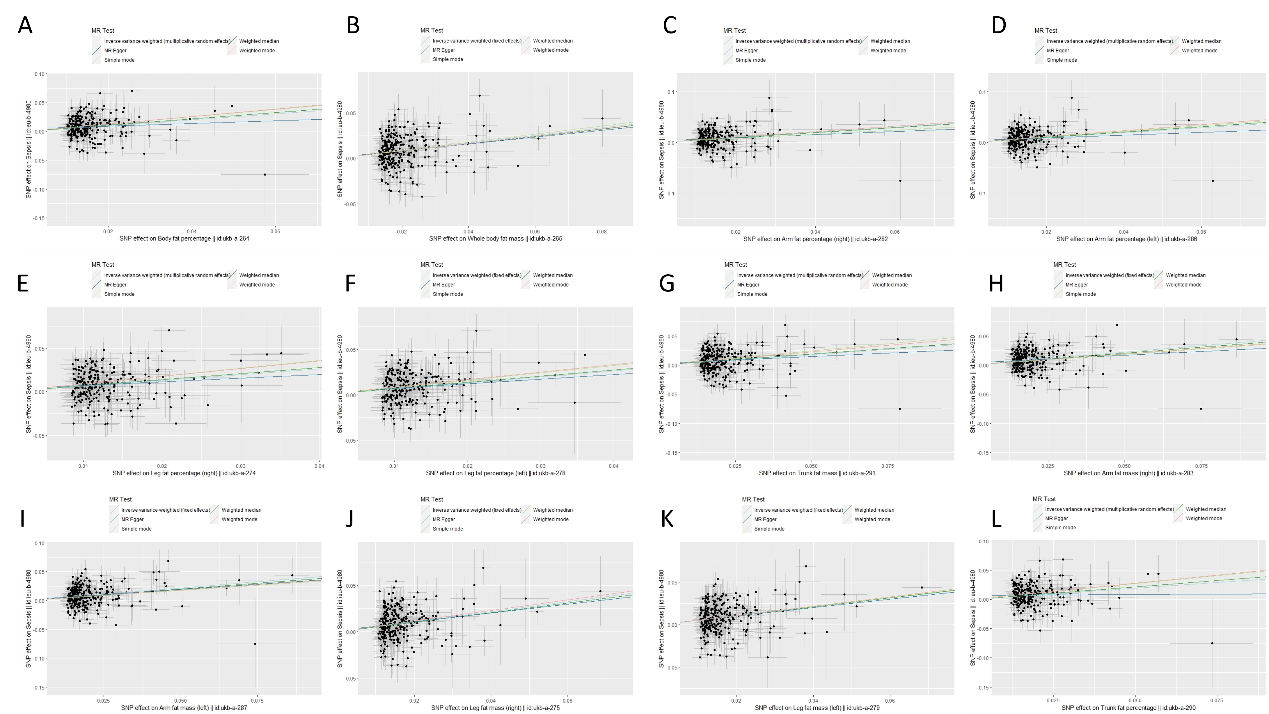


Supplementary Fig 4 Scatter plot showing the causality of fat mass on sepsis risk. (A) Causal effect of body fat percentage on sepsis risk. (B) Causal effect of Body fat mass on sepsis risk. (C) Causal effect of arm fat percentage (right) on sepsis risk. (D) Causal effect of arm fat percentage (left) on sepsis risk. (E) Causal effect of leg fat percentage (right) on sepsis risk. (F) Causal effect of leg fat percentage (left) on sepsis risk. (G) Causal effect of trunk fat mass on sepsis risk. (H) Causal effect of arm fat mass (right) on sepsis risk. (I) Causal effect of arm fat mass (left) on sepsis risk. (G) Causal effect of leg fat mass (right) on sepsis risk. (K) Causal effect of leg fat mass (left) on sepsis risk. (L) Causal effect of trunk fat percentage on sepsis risk. IVW, inverse-variance weighted; MR, Mendelian randomization.


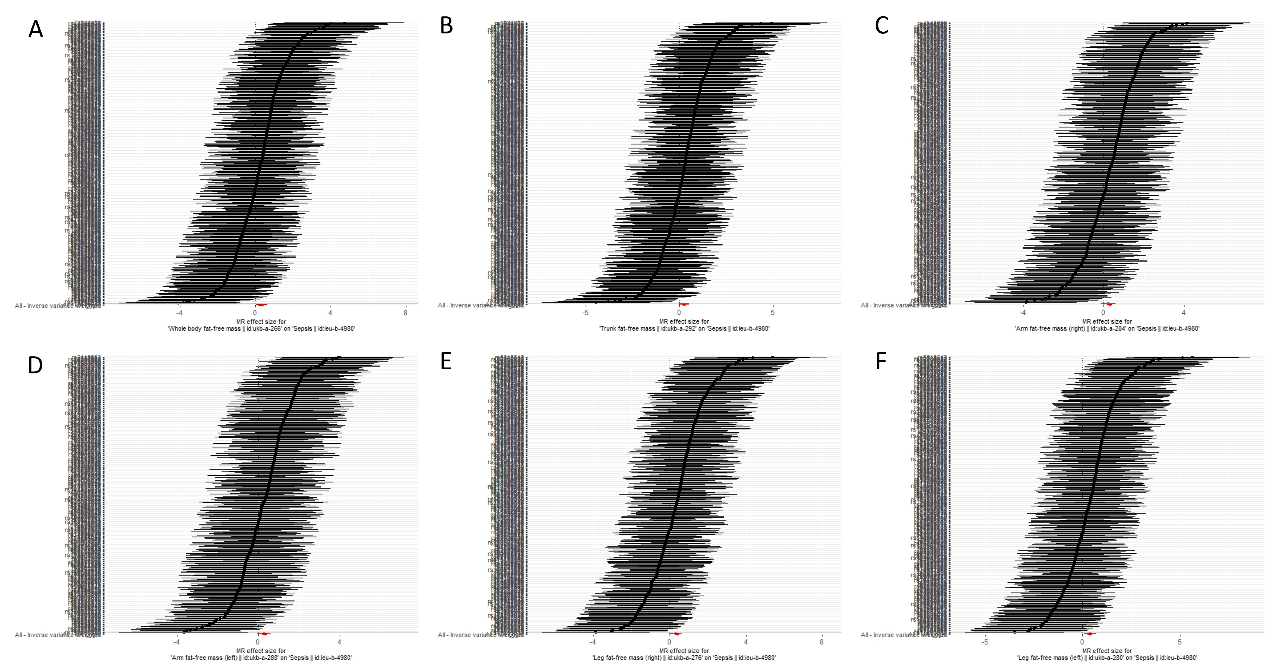


Supplementary Fig 5 Forest plot to visualize causal effect of each SNP on sepsis risk. (A) Causal effect of body nonfat mass on sepsis risk. (B) Causal effect of trunk nonfat mass on sepsis risk. (C) Causal effect of arm nonfat mass (right) on sepsis risk. (D) Causal effect of arm nonfat mass (left) on sepsis risk. (E) Causal effect of leg nonfat mass (right) on sepsis risk. (F) Causal effect of leg nonfat mass (left) on sepsis risk. IVW, inverse-variance weighted; MR, Mendelian randomization.


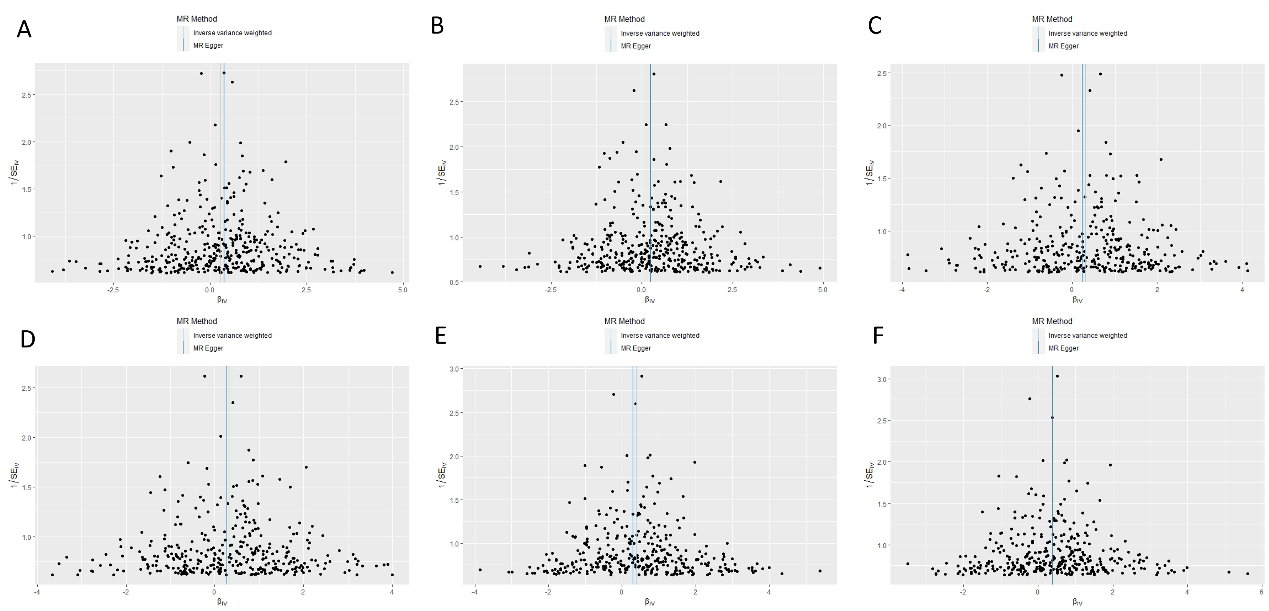


Supplementary Fig 6 Funnel plots to visualize overall heterogeneity of MR estimates for the effect of nonfat mass on sepsis risk. (A) Causal effect of body nonfat mass on sepsis risk. (B) Causal effect of trunk nonfat mass on sepsis risk. (C) Causal effect of arm nonfat mass (right) on sepsis risk. (D) Causal effect of arm nonfat mass (left) on sepsis risk. (E) Causal effect of leg nonfat mass (right) on sepsis risk. (F) Causal effect of leg nonfat mass (left) on sepsis risk. IVW, inverse-variance weighted; MR, Mendelian randomization.


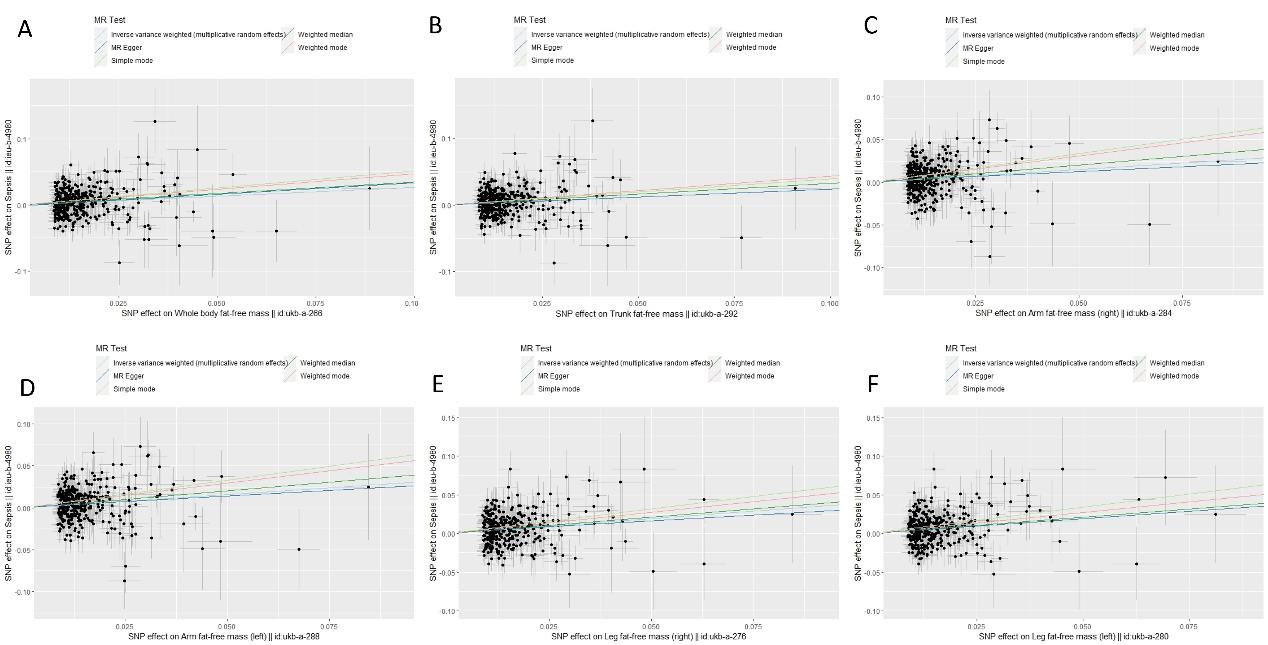


Supplementary Fig 7 Scatter plot showing the causality of nonfat mass on sepsis risk. (A) Causal effect of body nonfat mass on sepsis risk. (B) Causal effect of trunk nonfat mass on sepsis risk. (C) Causal effect of arm nonfat mass (right) on sepsis risk. (D) Causal effect of arm nonfat mass (left) on sepsis risk. (E) Causal effect of leg nonfat mass (right) on sepsis risk. (F) Causal effect of leg nonfat mass (left) on sepsis risk. IVW, inverse-variance weighted; MR, Mendelian randomization.


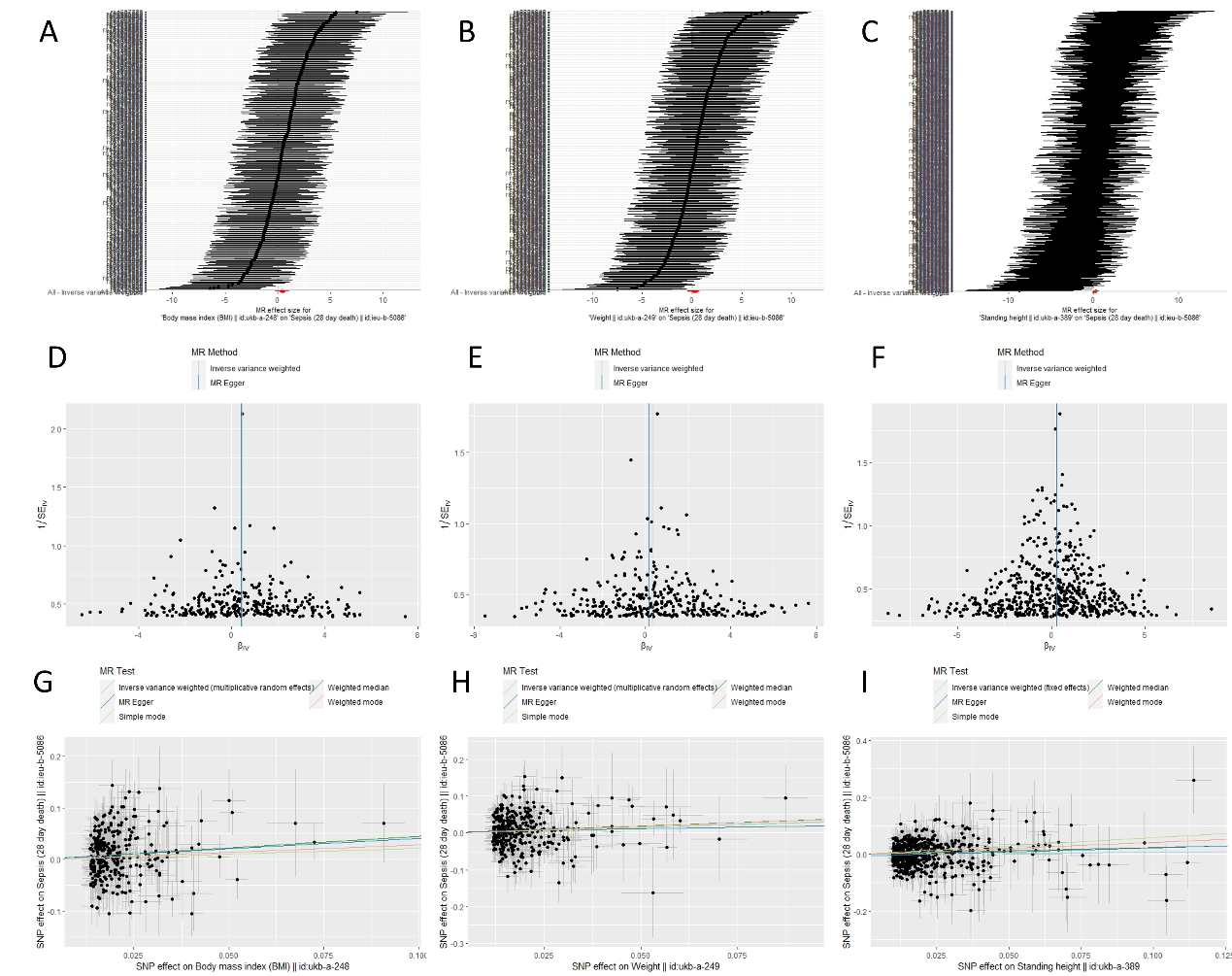


Supplementary Fig 8 The visualization for the causal effect of general anthropometric measures (BMI, weight and height) on sepsis mortality. (A-C) Forest plot to visualize causal effect of each SNP on sepsis risk. (A) Causal effect of BMI on sepsis risk. (B) Causal effect of weight on sepsis risk. (C) Causal effect of height on sepsis risk. (D-F) Funnel plots to visualize overall heterogeneity of MR estimates for the effect of BMI, weight and height on sepsis risk. (D) Causal effect of BMI on sepsis risk. (E) Causal effect of weight on sepsis risk. (F) Causal effect of height on sepsis risk. (G-I) Scatter plot showing the causality of BMI, weight and height on sepsis risk. (G) Causal effect of BMI on sepsis risk. (H) Causal effect of weight on sepsis risk. (I) Causal effect of height on sepsis risk. IVW, inverse-variance weighted; MR, Mendelian randomization.


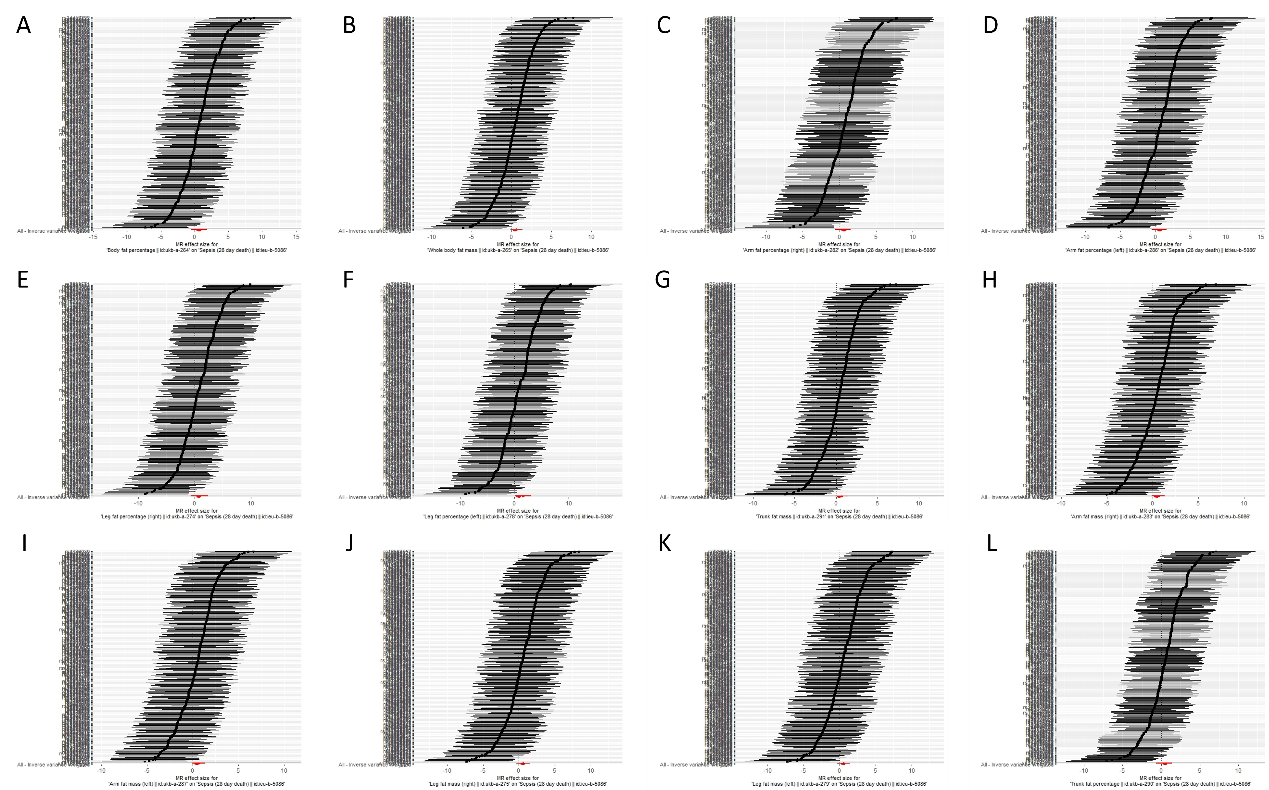


Supplementary Fig 9 Forest plot to visualize causal effect of each SNP on sepsis mortality. (A) Causal effect of body fat percentage on sepsis mortality. (B) Causal effect of Body fat mass on sepsis mortality. (C) Causal effect of arm fat percentage (right) on sepsis mortality. (D) Causal effect of arm fat percentage (left) on sepsis mortality. (E) Causal effect of leg fat percentage (right) on sepsis mortality. (F) Causal effect of leg fat percentage (left) on sepsis mortality. (G) Causal effect of trunk fat mass on sepsis mortality. (H) Causal effect of arm fat mass (right) on sepsis mortality. (I) Causal effect of arm fat mass (left) on sepsis mortality. (G) Causal effect of leg fat mass (right) on sepsis mortality. (K) Causal effect of leg fat mass (left) on sepsis mortality. (L) Causal effect of trunk fat percentage on sepsis mortality. IVW, inverse-variance weighted; MR, Mendelian randomization.


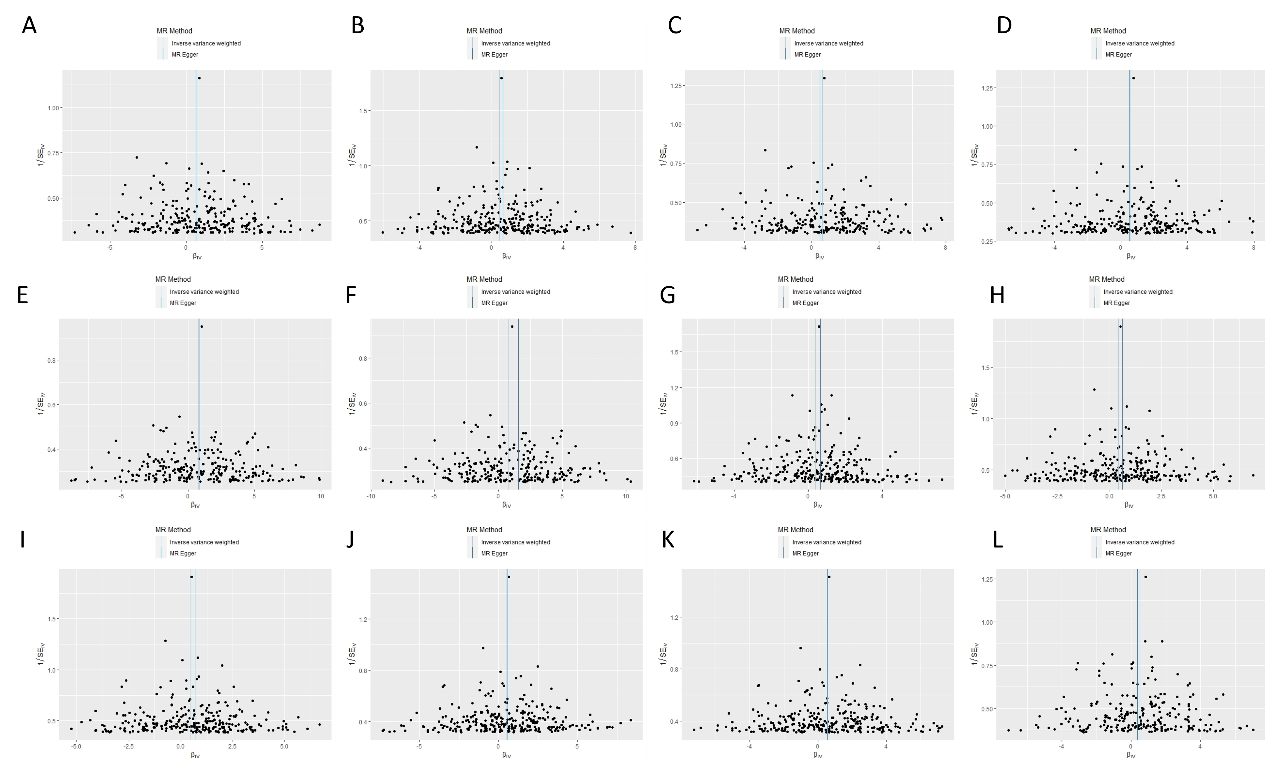


Supplementary Fig 10 Funnel plots to visualize overall heterogeneity of MR estimates for the effect of fat mass on sepsis mortality. (A) Causal effect of body fat percentage on sepsis mortality. (B) Causal effect of Body fat mass on sepsis mortality. (C) Causal effect of arm fat percentage (right) on sepsis mortality. (D) Causal effect of arm fat percentage (left) on sepsis mortality. (E) Causal effect of leg fat percentage (right) on sepsis mortality. (F) Causal effect of leg fat percentage (left) on sepsis mortality. (G) Causal effect of trunk fat mass on sepsis mortality. (H) Causal effect of arm fat mass (right) on sepsis mortality. (I) Causal effect of arm fat mass (left) on sepsis mortality. (G) Causal effect of leg fat mass (right) on sepsis mortality. (K) Causal effect of leg fat mass (left) on sepsis mortality. (L) Causal effect of trunk fat percentage on sepsis mortality. IVW, inverse-variance weighted; MR, Mendelian randomization.


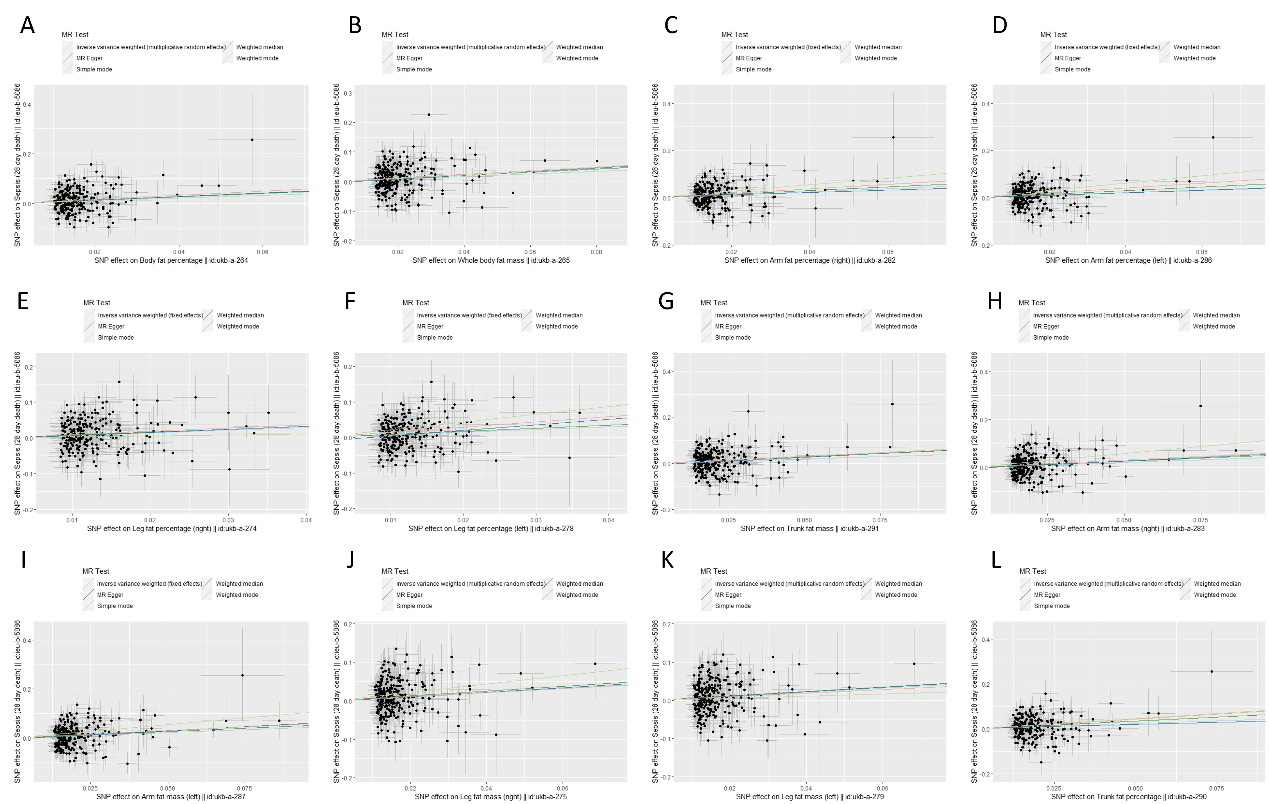


Supplementary Fig 11 Scatter plot showing the causality of fat mass on sepsis mortality. (A) Causal effect of body fat percentage on sepsis mortality. (B) Causal effect of Body fat mass on sepsis mortality. (C) Causal effect of arm fat percentage (right) on sepsis mortality. (D) Causal effect of arm fat percentage (left) on sepsis mortality. (E) Causal effect of leg fat percentage (right) on sepsis mortality. (F) Causal effect of leg fat percentage (left) on sepsis mortality. (G) Causal effect of trunk fat mass on sepsis mortality. (H) Causal effect of arm fat mass (right) on sepsis mortality. (I) Causal effect of arm fat mass (left) on sepsis mortality. (G) Causal effect of leg fat mass (right) on sepsis mortality. (K) Causal effect of leg fat mass (left) on sepsis mortality. (L) Causal effect of trunk fat percentage on sepsis mortality. IVW, inverse-variance weighted; MR, Mendelian randomization.


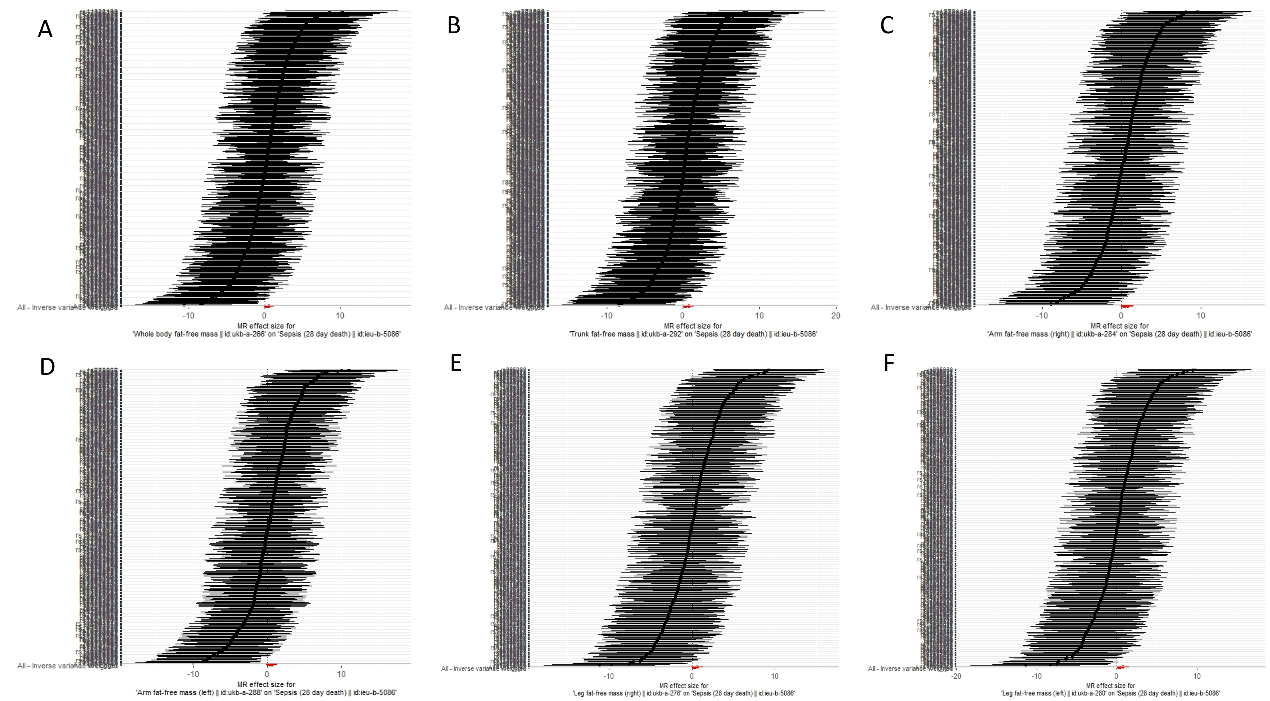


Supplementary Fig 12 Forest plot to visualize causal effect of each SNP on sepsis mortality. (A) Causal effect of body nonfat mass on sepsis mortality. (B) Causal effect of trunk nonfat mass on sepsis mortality. (C) Causal effect of arm nonfat mass (right) on sepsis mortality. (D) Causal effect of arm nonfat mass (left) on sepsis mortality. (E) Causal effect of leg nonfat mass (right) on sepsis mortality. (F) Causal effect of leg nonfat mass (left) on sepsis mortality. IVW, inverse-variance weighted; MR, Mendelian randomization.


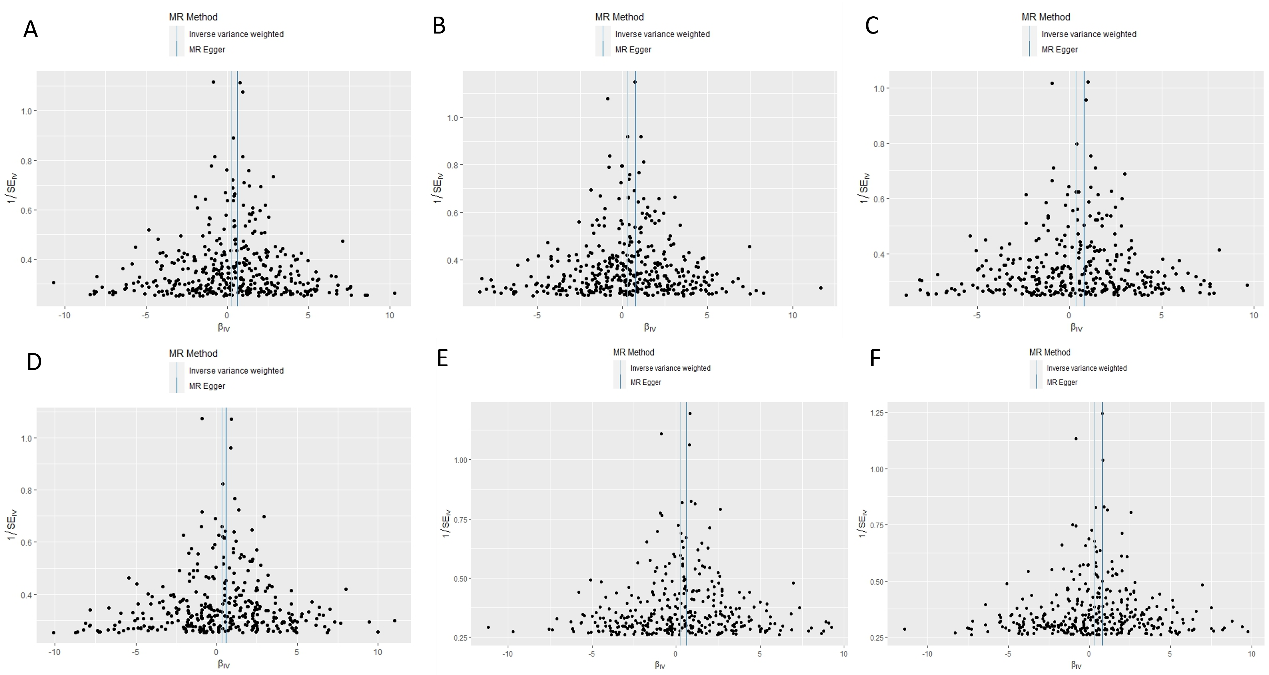


Supplementary Fig 13 Funnel plots to visualize overall heterogeneity of MR estimates for the effect of nonfat mass on sepsis mortality. (A) Causal effect of body nonfat mass on sepsis mortality. (B) Causal effect of trunk nonfat mass on sepsis mortality. (C) Causal effect of arm nonfat mass (right) on sepsis mortality. (D) Causal effect of arm nonfat mass (left) on sepsis mortality. (E) Causal effect of leg nonfat mass (right) on sepsis mortality. (F) Causal effect of leg nonfat mass (left) on sepsis mortality. IVW, inverse-variance weighted; MR, Mendelian randomization.


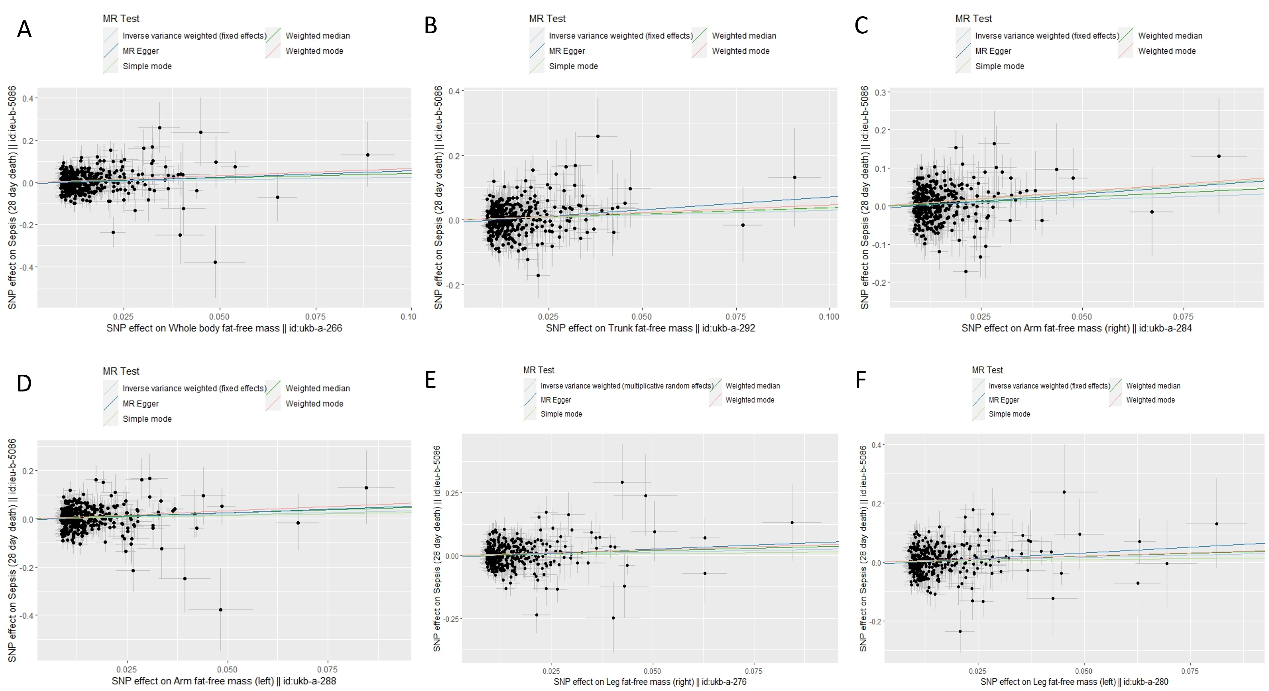


Supplementary Fig 14 Scatter plot showing the causality of nonfat mass on sepsis mortality. (A) Causal effect of body nonfat mass on sepsis mortality. (B) Causal effect of trunk nonfat mass on sepsis mortality. (C) Causal effect of arm nonfat mass (right) on sepsis mortality. (D) Causal effect of arm nonfat mass (left) on sepsis mortality. (E) Causal effect of leg nonfat mass (right) on sepsis mortality. (F) Causal effect of leg nonfat mass (left) on sepsis mortality. IVW, inverse-variance weighted; MR, Mendelian randomization.


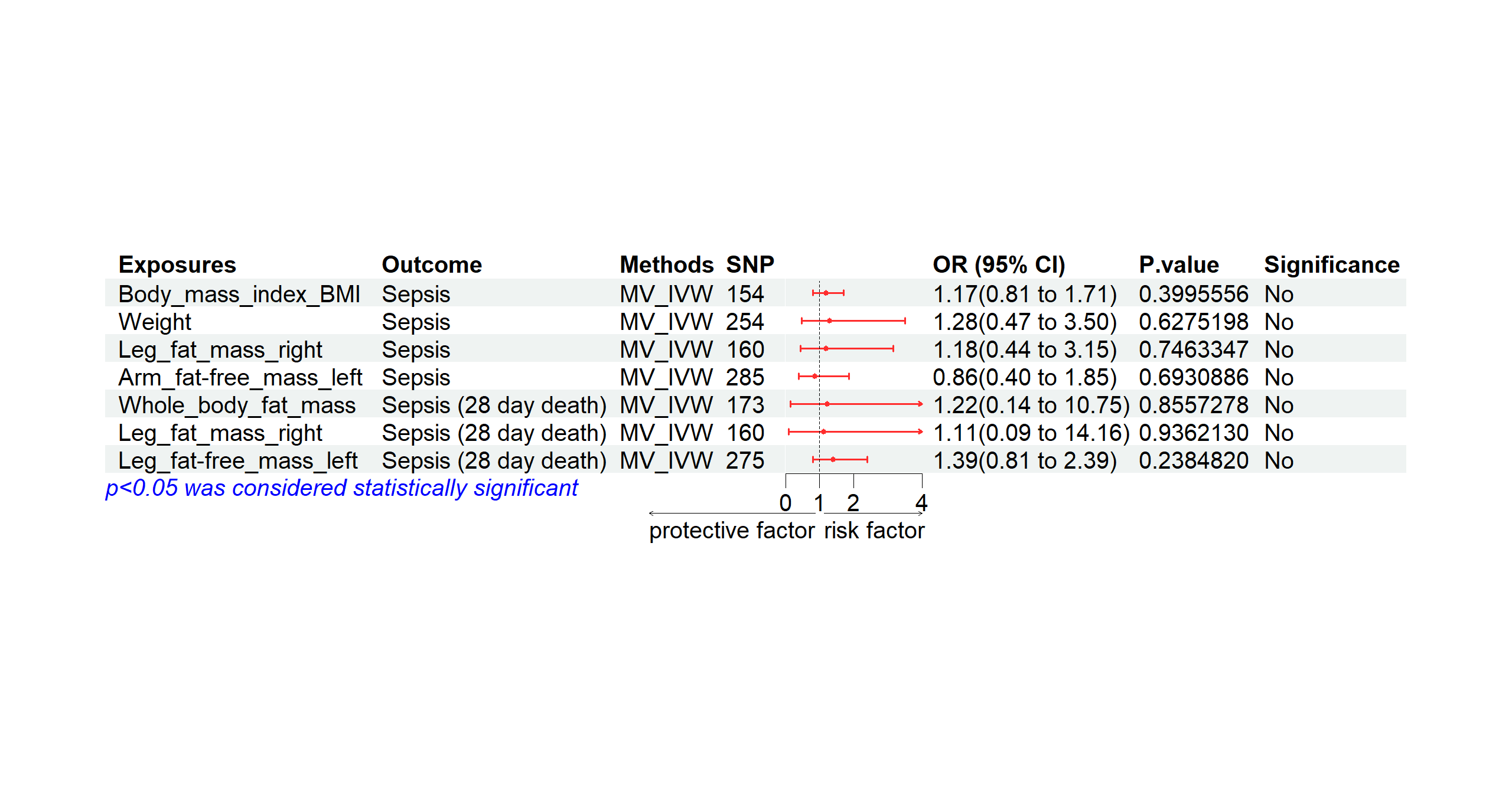


Supplementary Fig 15 Multivariable MR (MVMR) revealing the associations of anthropometric measures with the sepsis risk and mortality after lasso regression screening. CI, confidence interval; IVW, inverse variance weighting; SNP: number of single-nucleotide polymorphisms that included in the analysis; OR, odds ratio.
