# Supplementary material for: Mendelian randomization analysis reveals causal association of anthropometric measures on sepsis risk and mortality
Source: PLoS One. 2024 Sep 30;19(9):e0310898. doi: 10.1371/journal.pone.0310898 (PMC11441680; doi:10.1371/journal.pone.0310898)
Supplement: Attachment — Submitted filename: Responses_to_Reviewers.docx [file pone.0310898.s005.docx]

**Reviewers' Comments:**

**General response:** We thank the reviewers for their time and insightful comments. We have carefully considered these comments and substantially revised the manuscript by thoroughly researching the field reviewed and clarifying the content of this manuscript to make it more comprehensive. Below, we address each of the reviewers’ questions.

**Reviewer 1**

***I would like to commend you for your work on investigating the causal relationship between anthropometric measures and sepsis risk. It addresses a significant question with the potential for important clinical implications. However, after a thorough review, several aspects of the manuscript require further clarification and expansion.***

**Our response:** We appreciate the reviewer’s critical and constructive comments. It is our honour to get your help to revise our manuscript. Your comments give us valuable assistance in improving our manuscript. We have carefully revised our manuscript accordingly. After adopting your suggestions, we believe a significant improvement can be seen in our revised manuscript. The following are detailed responses to your recommendations.

**1. *Introduction/Discussion: The introduction and discussion sections of your paper seem somewhat vague. The depth of discussion could be improved to better align with the results. While the introduction primarily discusses the contrasting findings of BMI and obesity related to sepsis risk, the main results appear to focus on fat mass parameters. It would be beneficial if these aspects were more thoroughly addressed and integrated into the research aims and background.***

**Our response:** Thank you for your feedback and comments regarding the vagueness of the introduction and discussion sections. We are very grateful that the reviewer read our article carefully. As we all know, currently, BMI, the most common fat mass parameters, is commonly used to identify obese persons due to its ease of calculation and cost-effectiveness. However, its use has been repeatedly criticized for its shortcomings. For example, BMI does not distinguish between fat mass and nonfat mass, and dose not capture body fat location, but different types of tissue mass and different fat location have been demonstrated to play different role in the development of disease. As precision medicine continue developing, it is necessary to investigate and determine the specific effect of different types of tissue mass and different fat location on disease. Thus, this study was aimed at increasing the understanding of the obesity-sepsis association by evaluating the causal effects of fat mass, nonfat mass and height (henceforth, ‘anthropometric measures’) on sepsis risk and mortality via a MR study using results from large genome-wide association studies (GWAS). On the other hand, the tissue mass of different types and locations can be reflected by detailed fat mass parameters. That’s why the main results in this study appear to focus on fat mass parameters. Undoubtedly, your suggestion is of great help to improve the quality and preciseness of this manuscript. Hence, we have made some revisions, which was showed in red.

***2. Results: There appears to be an error in the reporting of results on lines 177-178. The statement reads: "Weighted median method also supported the intimated association between nonfat mass and the risk of sepsis (all p>0.05)". Based on the Mendelian Randomization theory and the figures presented in Fig 3, the p-values estimated by the weighted median method should all be less than 0.05.***

**Our response:** It’s our negligence for this mistake. We have revised this error. Thanks very much for your careful review again. Even so, we are still really sorry for our cursoriness. **Please see Page 8-9, *Lines 172-174.***

***(Lines 172-174):***

The results from Weighted median method also supported the intimate association between nonfat mass and the risk of sepsis (all p < 0.05).

***3. Interpretation of Cochrane's Q tests and MR-Egger Intercept tests: The interpretation of these tests seems to lack sufficient discussion. Kindly provide explanations for potential reasons that could lead to a significant Cochrane's Q test and an insignificant MR-Egger intercept test.***

**Our response:** We appreciate your good suggestion on improving the accuracy of the expression. The potential cause of the heterogeneity is probably involved in instrumental variables from different analysis platforms, experiments, populations, etc., The significant heterogeneity will affect the results of Mendelian randomization analysis. Thus, we usually need to evaluate the heterogeneity of genetic variant using Cochran’s Q test at first. If there is no excess heterogeneity in the variant-specific causal estimates, then the random-effects and fixed-effect results will be identical. So, there is no loss of precision. If there is excess heterogeneity in the variant specific causal estimates, then the fixed-effect estimate is overly precise, and this heterogeneity should be accounted for. **If there is statistical evidence for a causal effect in a random-effects analysis, this means that there is consistent evidence that the genetic variants support a causal effect of the exposure on the outcome even accounting for heterogeneity in the variant-specific causal estimates.** Thus, in this study, if there is heterogeneity (P-value≤0.05), random-effects IVW models are applied; otherwise (P-value>0.05), the fixed-effect IVW model is applied.

On the other hand, in order to evaluate whether instrumental variables influence outcomes through factors other than exposure, pleiotropic analysis was performed using MR-Egger intercept test in this study. The results did not reveal any evidence of horizontal pleiotropy during the MR analyses, as all p values were above 0.05.

However, it’s our negligence for the insufficient discussion of the interpretation of these tests. Thus, we supplemented it in our manuscript. **Please see Page 12, *Lines 229-241.***

***(Lines 229-241):***

**Table 1** displays the findings from Cochrane’s Q test and pleiotropy test. Specifically, our analysis suggested that potential heterogeneity was observed in the MR analyses of some anthropometric measures and sepsis. The significant heterogeneity will affect the results of Mendelian randomization analysis. Thus, in this study, if there is no heterogeneity (P-value>0.05), the fixed-effect IVW model was applied; otherwise (P-value≤0.05), random-effects IVW model was applied. If there is statistical evidence for a causal effect in a random-effects analysis, this means that there is consistent evidence that the genetic variants support a causal effect of the exposure on the outcome even accounting for heterogeneity in the variant-specific causal estimates. Nevertheless, the MR-Egger intercept tests did not reveal any evidence of horizontal pleiotropy during the MR analyses, as all p values were above 0.05. Taken together, all of the findings suggest that the association between genetic predisposition to anthropometric measures and sepsis was significantly impacted by any individual SNP.

***4. Interrelationship between Anthropometric Measures: It would be helpful if you could discuss how you addressed the interrelationship between anthropometric measures, which could introduce bias. If the exposures are highly correlated (i.e., collinear), it becomes challenging to isolate the causal effects of individual exposures. This is due to the possibility of genetic variants used as instrumental variables (IVs) for one exposure also being associated with other exposures—a problem further compounded in cases of multicollinearity.***

**Our response:** Thanks very much for your constructive suggestions. You are right, if the exposures are highly correlated (i.e., collinear), it becomes challenging to isolate the causal effects of individual exposures. Thus, we performed multivariable MR to evaluate the independent causal effects of relevant anthropometric measures on sepsis outcomes. However, the positive results of MVMR showed very wide 95%CI of OR, which indicating the multicollinearity among anthropometric measures. Thus, lasso regression analysis was used to remove highly correlated exposures. In lasso regression model, four anthropometric measures (BMI, weight, leg fat mass (right) and arm fat-free mass (left)) were selected for sepsis risk evaluation and three anthropometric measures (body fat mass, leg fat mass (right) and leg fat-free mass (left)) for sepsis mortality evaluation. The results revealed that compared with aforementioned estimates, no causal association was observed between these selected anthropometric measures and sepsis (Supplementary Fig 15). Thus, the observed effects for weight-related measures in the univariable MR analyses are more likely a bias caused by the interrelationship between anthropometric measures. These findings probably provide a novel understanding for the reason why “obesity paradox” occur in sepsis: namely previous literatures only paid attention to the association between BMI and sepsis and did not consider the collinear effect from other anthropometric measures. **Please see Page 5, *Lines 115-121;* Page 11-12, *Lines 206-227;* Page 15*, Lines 293-300.***

***(Lines 115-121):***

Importantly, multivariable MR (MVMR) was next conducted to evaluate the independent causal effects of relevant anthropometric measures on sepsis outcomes. This method is an effective statistical approach, enabling us to estimate the direct effect of each phenotype on the outcome by incorporating SNPs with multiple phenotypes into the analysis^1^. Here, the least absolute shrinkage and selection operator (LASSO) regression was used to remove highly correlated exposures in the presence of various exposures, enhancing the accuracy and reliability of the results^2^.

***(Lines 204-226):***

For the MVMR analyses, we constructed a model with anthropometric measures that retained an effect on sepsis risk and mortality in the univariable MR models. Supplementary table 2 and 3 present the comprehensive estimated values and confidence intervals for the model included in the multivariable analysis. The positive results of MVMR, after accounting for various types of anthropometric measures, were as follows: arm fat percentage (left) and sepsis [OR (95% CI): 0.002(0.000–0.549), P = 0.03]; body fat mass and sepsis mortality [OR (95% CI): 1.39 x10^^5^ (2.745–7.023 x10^^7^), P = 0.03]; leg fat percentage (left) and sepsis mortality [OR (95% CI): 2.061x10^^7^ (1.580–2.69x10^^14^), P = 0.04]; leg fat mass (left) and sepsis mortality [OR (95% CI): 0.000(0.000–0.667), P < 0.05]; leg fat-free mass (left) and sepsis mortality [OR (95% CI): 1.825 x10^^3^ (5.231–6.367 x10^^5^), P = 0.01]; arm fat percentage (left) and sepsis mortality [OR (95% CI): 0.000(0.000–0.050), P = 0.02] (Supplementary table 2-3). Obviously, the wide 95%CI of OR indicated the multicollinearity among anthropometric measures. Thus, lasso regression analysis was used to remove highly correlated exposures. In lasso regression model, four anthropometric measures (BMI, weight, leg fat mass (right) and arm fat-free mass (left)) were selected for sepsis risk evaluation and three anthropometric measures (body fat mass, leg fat mass (right) and leg fat-free mass (left)) for sepsis mortality evaluation. The results revealed that compared with aforementioned estimates, no causal association was observed between these selected anthropometric measures and sepsis (Supplementary Fig 15). Thus, the observed effects for anthropometric measures in the univariable MR analyses are more likely operating through the pathways of other anthropometric measures.

***(Lines 293-300):***

Thus, we further performed MVMR analyses to assess the independent causal effects of BMI on sepsis risk utilizing lasso regression method. Our results demonstrated that the direct estimates for the BMI were significantly attenuated, causing a wider 95% CI that overlapped null. The same phenomenon was also seen in other anthropometric measures displaying significant causality with sepsis in univariable MR analysis. Hence, the observed effects for BMI in the univariable MR analyses are more likely a bias caused by the interrelationship between anthropometric measures.

***5. Same Population for Exposure and Outcome Data: Using the same population for all exposure and outcome data can introduce sample overlap bias. Please address this potential bias in your methodology or discussion.***

**Our response:** We are very grateful that the reviewer read our article carefully and has provided this idea. The inclusion of the same population for all exposure and outcome data can help us obtain the most reliable results. That is what's actually done in many other literatures^3^^,^ ^4^. However, from another perspective you are right, because the results obtained from the same population for all exposure and outcome data probably are inapplicable to other population. Thus, the relationship between anthropometric measures and sepsis revealed in this study need to be further demonstrated through including different population. This is undoubtedly a limitation of this study. Hence, we have supplemented this limitation in our discussion. **Please see Page 16, *Lines 320-324.***

***(Lines 320-324):***

Firstly, only European populations were included. However, different cohorts of patients have different genetic profiles due to the difference of ethnic diversity, risk factors, and epigenetic profiles. Thus, the reliability of the causal relationships from our analysis should be demonstrated in other non-European descent.

***6. In conclusion, your research addresses an important topic, and with some additional work, it could provide valuable insights into the study of sepsis risk. I look forward to your response and the subsequent version of your manuscript.***

**Our response:** Thanks for your recognition.

**Reviewer #2:**

***The authors performed a mendelian randomization (MR) study to investigate the link between anthropometric traits such as weight, BMI, height, etc., and sepsis and sepsis death. The manuscript lacks a detailed description of the methods used and plots that are normally included in MR studies (see comments below).***

**Our response:** We appreciate the reviewer’s critical and constructive comments. It is our honour to get your help to revise our manuscript. Your comments give us valuable assistance in improving our manuscript. We have carefully revised our manuscript accordingly. After adopting your suggestions, we believe a significant improvement can be seen in our revised manuscript. The following are detailed responses to your recommendations.

***1. - This is basically a one-sample MR since both the GWAS on the exposure and that on the outcome came from the same cohort (UK biobank). As this approach possess some limitations, these should be discussed in the text. Especially because two-samples MR studies on BMI and sepsis already exists.***

**Our response:** We are very grateful for your review of our article carefully and for providing useful ideas. We agree with your view. Thus, we have supplemented this limitation in our discussion. **Please see Page 16, *Lines 320-324.*** On the other hand, there were indeed two-samples MR studies reported the relationship between BMI and sepsis. For example, Wang et al in 2023 indicated that their two-sample Mendelian randomization supports a causal relationship between BMI and sepsis, and thus further concluded that proper control of BMI may prevent sepsis^5^. However, BMI has received criticism recently for the following reasons^6^: 1) BMI falls short in its ability to distinguish between fat mass and non-fat mass (e.g., lean tissue mass), which might lead to the incorrect assessments of certain individuals. 2) BMI does not display the specific location of body fat which has been demonstrated to exert a vital role in forecasting the risk of several diseases. That’s why this study was designed to increase the understanding of the obesity-sepsis association by evaluating the causal effects of fat mass, nonfat mass and height on sepsis risk and mortality.

***(Lines 320-324):***

Firstly, only European populations were included. However, different cohorts of patients have different genetic profiles due to the difference of ethnic diversity, risk factors, and epigenetic profiles. Thus, the reliability of the causal relationships from our analysis should be demonstrated in other non-European descent.

***2. - The methods used for LD clumping and MR should be described and the relevant references and software cited.***

**Our response:** It’s our negligence to omit this important information. We have supplemented the description regarding the methods used for LD clumping and MR, and the relevant references and software cited were also added in this manuscript. **Please see Page 4-5, *Lines 92-94;* Page 6, *Lines 129-130.***

***(Lines 92-94):***

Then we harmonized these selected variants with sepsis data, excluding palindromic SNPs and clumping for linkage disequilibrium (LD, a window size greater than 10,000 kb, r^2^<0.001)^7^.

***(Lines 129-130):***

In this study, all of MR analysis were performed by R software (version 4.2.0) and TwoSample MR package (version 0.5.6).

***3. - Methods: "The weighted median yielded consistent estimates even when over 50% of genetic SNPs have horizontal pleiotropy" This is a little ambiguous, I would rephrase it with something like "The weighted median requires that at least the 50% of the weight come from valid IVs".***

**Our response:** We appreciate your great suggestion on improving the accessibility of our manuscript. We have revised this sentence according to your suggestion. **Please see Page 5, *Lines 109-110.***

***(Lines 109-110):***

The weighted median requires that at least the 50% of the weight come from valid IVs^8^

***4.*** ***- The last sentence of the methods mentions a correction for multiple tests. However, it is not clear how and where it was applied.***

**Our response:** We are sorry that we were not clear enough in this section to mislead you. We have revised these contents in our manuscript. **Please see Page 7, *Lines 146-147;* Page 8, *Lines 159-161;* Page 8-9, *Lines 172-174;* Page 12, *Lines 229-241.***

***(Lines 146-147):***

Weighted median method also confirmed these associations between general anthropometric measures and sepsis risk.

***(Lines 159-161):***

These results were demonstrated by Weighted median method, indicating that the anthropometric measures of fat mass had significantly causal MR association with the risk of risk with all p-values less than 0.05.

***(Lines 172-174):***

The results from Weighted median method also supported the intimate association between nonfat mass and the risk of sepsis (all p < 0.05).

***(Lines 229-241):***

**Table 1** displays the findings from Cochrane’s Q test and pleiotropy test. Specifically, our analysis suggested that potential heterogeneity was observed in the MR analyses of some anthropometric measures and sepsis. The significant heterogeneity will affect the results of Mendelian randomization analysis. Thus, in this study, if there is no heterogeneity (P-value>0.05), the fixed-effect IVW model was applied; otherwise (P-value≤0.05), random-effects IVW model was applied. If there is statistical evidence for a causal effect in a random-effects analysis, this means that there is consistent evidence that the genetic variants support a causal effect of the exposure on the outcome even accounting for heterogeneity in the variant-specific causal estimates. Nevertheless, the MR-Egger intercept tests did not reveal any evidence of horizontal pleiotropy during the MR analyses, as all p values were above 0.05. Taken together, all of the findings suggest that the association between genetic predisposition to anthropometric measures and sepsis was significantly impacted by any individual SNP.

***5. - The results contain long lists of the estimated OR, CI and p-value that are already reported in the tables and that make the text hard to read***

**Our response:** We are sorry for the unapt and redundant expression mode in the results. We have revised and deleted these contents pointed by you.

***6. - Results line 178: "(all p > 0.05 )" should probably be "(all p < 0.05)".***

**Our response:** It’s our negligence for this mistake. We have revised this error. Thanks very much for your careful review again. Even so, we are still really sorry for our cursoriness. **Please see Page 8-9, *Lines 172-174.***

***(Lines 172-174):***

The results from Weighted median method also supported the intimate association between nonfat mass and the risk of sepsis (all p < 0.05).

***7. - All tables are reported as figures, which make them difficult to read. Moreover, a table with the markers used for the analyses could be reported in supplementary tables.***

**Our response:** We appreciate your great suggestion on improving the accessibility of our manuscript. You are right, it’s difficult to read and understand when all results are reported as figures. Thus, we displayed the main MR results using forest plots **(Please see Figure 1; other plots were presented in the same way)**. In addition, the data in Table 1 in our previous manuscript was moved to supplementary table 1. We appreciate your time and consideration, and we remain open to any further suggestions or feedback you may have.

**Fig 1** **Associations of general anthropometric measures with the risk of sepsis.** CI, confidence interval; IVW, inverse variance weighting; SNP: number of single-nucleotide polymorphisms that included in the analysis; OR, odds ratio.

***8. - It is common practice to display the results of MRs by a scatterplot or other plots. Also, consider to include leave-one-out forest plots, at least for the main analyses.***

**Our response:** Thanks very much for your constructive suggestions. We agree with the reviewer's point of view. We have displayed the results of MRs by a scatterplot, forest plot, and funnel plot, and supplemented these figures in our supplementary material. **Please see Page 7, *Lines 138-140;***

***(Lines 138-140):***

The causal effects of each genetic variant on sepsis are shown via scatterplot, funnel plot and scatter plot, which were summarized in Supplementary Figures 1-14.

***9. - In the discussion, the potential role of obesity and BMI on sepsis is discussed. As other studies already showed an association between BMI and sepsis by two-sample MR, the authors could elaborate on the association between non-fat mass and sepsis.***

**Our response:** We appreciate your great suggestion on improving the accessibility of our manuscript. We have supplemented the discussion regarding the association between non-fat mass and sepsis in our manuscript. **Please see Page 16, *Lines 309-319.***

***(Lines 309-319):***

It is worth mentioning that the association of lean mass (namely nonfat mass, mainly including muscle and bone) change with sepsis has also been revealed in recent literature. It’s reported that sepsis can induce a series of metabolic changes and then further lead to obvious nonfat mass loss to provide enough energy for body metabolism^9^. These metabolic disorders prevent survival patients from recovering to regular daily activities and are directly involving in poor prognosis. For example, Lee et al revealed that muscle Mass loss/depletion was associated with increased sepsis mortality^10^. However, it remains unclear whether the demonstrated associations are causal. In order to reveal this mystery, we performed this MR analysis and found that genetically mediated nonfat mass is causally associated with an increased risk of sepsis.

***10. - Although I am not an expert of the field, I think it might be worth to discuss the possible effect of*** ***confounders on the relationship between BMI and mortality due to sepsis since BMI has been linked to mortality in many ways.***

**Our response:** Thank you for your suggestion on improving the readability of our manuscript. In order to eliminate the effect of confounders on the relationship between BMI and mortality, multivariable MR (MVMR) was performed. In MVMR analysis, we found that the casual association of BMI with sepsis revealed in univariable MR was removed. Thus, the observed effects for BMI in the univariable MR analyses are more likely a bias caused by the interrelationship between anthropometric measures. These findings probably provide a novel understanding for the reason why “obesity paradox” occur in sepsis: namely previous literatures only paid attention to the association between BMI and sepsis and did not consider the collinear effect from other anthropometric measures. **Please see Page 5, *Lines 115-121;* Page 11-12, *Lines 206-227;* Page 15*, Lines 293-300;* Page 17, *Lines 336-344.***

***(Lines 115-121):***

Importantly, multivariable MR (MVMR) was next conducted to evaluate the independent causal effects of relevant anthropometric measures on sepsis outcomes. This method is an effective statistical approach, enabling us to estimate the direct effect of each phenotype on the outcome by incorporating SNPs with multiple phenotypes into the analysis^1^. Here, the least absolute shrinkage and selection operator (LASSO) regression was used to remove highly correlated exposures in the presence of various exposures, enhancing the accuracy and reliability of the results^2^.

***(Lines 206-227):***

For the MVMR analyses, we constructed a model with anthropometric measures that retained an effect on sepsis risk and mortality in the univariable MR models. Supplementary table 2 and 3 present the comprehensive estimated values and confidence intervals for the model included in the multivariable analysis. The positive results of MVMR, after accounting for various types of anthropometric measures, were as follows: arm fat percentage (left) and sepsis [OR (95% CI): 0.002(0.000–0.549), P = 0.03]; body fat mass and sepsis mortality [OR (95% CI): 1.39 x10^^5^ (2.745–7.023 x10^^7^), P = 0.03]; leg fat percentage (left) and sepsis mortality [OR (95% CI): 2.061x10^^7^ (1.580–2.69x10^^14^), P = 0.04]; leg fat mass (left) and sepsis mortality [OR (95% CI): 0.000(0.000–0.667), P < 0.05]; leg fat-free mass (left) and sepsis mortality [OR (95% CI): 1.825 x10^^3^ (5.231–6.367 x10^^5^), P = 0.01]; arm fat percentage (left) and sepsis mortality [OR (95% CI): 0.000(0.000–0.050), P = 0.02] (Supplementary table 2-3). Obviously, the wide 95%CI of OR indicated the multicollinearity among anthropometric measures. Thus, lasso regression analysis was used to remove highly correlated exposures. In lasso regression model, four anthropometric measures (BMI, weight, leg fat mass (right) and arm fat-free mass (left)) were selected for sepsis risk evaluation and three anthropometric measures (body fat mass, leg fat mass (right) and leg fat-free mass (left)) for sepsis mortality evaluation. The results revealed that compared with aforementioned estimates, no causal association was observed between these selected anthropometric measures and sepsis (Supplementary Fig 15). Thus, the observed effects for anthropometric measures in the univariable MR analyses are more likely operating through the pathways of other anthropometric measures.

***(Lines 293-300):***

Thus, we further performed MVMR analyses to assess the independent causal effects of BMI on sepsis risk utilizing lasso regression method. Our results demonstrated that the direct estimates for the BMI were significantly attenuated, causing a wider 95% CI that overlapped null. The same phenomenon was also seen in other anthropometric measures displaying significant causality with sepsis in univariable MR analysis. Hence, the observed effects for BMI in the univariable MR analyses are more likely a bias caused by the interrelationship between anthropometric measures.

***(Lines 336-344):***

In conclusion, the current work firstly provides evidence that weight-related measures have a causal association on sepsis risk and mortality via univariable MR analyses, but MVMR analysis indicated the observed effects for weight-related measures in the univariable MR analyses are more likely a bias caused by the interrelationship between anthropometric measures. This probably explain the reason why “obesity paradox” occur in sepsis: namely previous literatures only paid attention to the association between BMI and sepsis and did not consider the collinear effect from other anthropometric measures. Thus, these findings represent vital new knowledge on the role of anthropometric-related measures in the sepsis etiology.

***11. - Compliance with Ethics Guidelines: the authors state that the manuscript follows the STROBE-MR guidelines. I disagree on this point. The STROBE-MR paper contains a detailed table describing how to report the results of MR based studies. In my opinion, the manuscript is not structured according to such guidelines.***

**Our response:** We are very grateful for your review of our article carefully and for providing useful ideas. Indeed, the manuscript was not strictly structured according to such guidelines because some requirements are inapplicable to this study. But we included the crucial contents of STROBE-MR guidelines in our manuscript. In order to normatively and scientifically report our results of MR, we completed and supplemented the STROBE-MR checklist.

1.Sanderson E, Davey Smith G, Windmeijer F, et al. An examination of multivariable Mendelian randomization in the single-sample and two-sample summary data settings. Int J Epidemiol 2019;48:713-27. 10.1093/ije/dyy262.

2.Sun Z, Tao Y, Li S, et al. Statistical strategies for constructing health risk models with multiple pollutants and their interactions: possible choices and comparisons. Environ Health 2013;12:85. 10.1186/1476-069x-12-85.

3.Almramhi MM, Storm CS, Kia DA, et al. The role of body fat in multiple sclerosis susceptibility and severity: A Mendelian randomisation study. Mult Scler 2022;28:1673-84. 10.1177/13524585221092644.

4.Speed MS, Jefsen OH, Børglum AD, et al. Investigating the association between body fat and depression via Mendelian randomization. Transl Psychiatry 2019;9:184. 10.1038/s41398-019-0516-4.

5.Wang J, Hu Y, Zeng J, et al. Exploring the Causality Between Body Mass Index and Sepsis: A Two-Sample Mendelian Randomization Study. Int J Public Health 2023;68:1605548. 10.3389/ijph.2023.1605548.

6.Frankenfield DC, Rowe WA, Cooney RN, et al. Limits of body mass index to detect obesity and predict body composition. Nutrition 2001;17:26-30. 10.1016/s0899-9007(00)00471-8.

7.J B, J T, R G. The Rough Guide to Genes & Cloning. 2007.

8.Bowden J, Davey Smith G, Haycock PC, et al. Consistent Estimation in Mendelian Randomization with Some Invalid Instruments Using a Weighted Median Estimator. Genet Epidemiol 2016;40:304-14. 10.1002/gepi.21965.

9.Rosenthal MD, Moore FA. Persistent inflammatory, immunosuppressed, catabolic syndrome (PICS): A new phenotype of multiple organ failure. J Adv Nutr Hum Metab 2015;1. 10.14800/janhm.784.

10.Lee Y, Park HK, Kim WY, et al. Muscle Mass Depletion Associated with Poor Outcome of Sepsis in the Emergency Department. Ann Nutr Metab 2018;72:336-44. 10.1159/000488994.
