# Supplementary material for: Mendelian randomization analysis reveals causal association of anthropometric measures on sepsis risk and mortality
Source: PLoS One. 2024 Sep 30;19(9):e0310898. doi: 10.1371/journal.pone.0310898 (PMC11441680; doi:10.1371/journal.pone.0310898)
Supplement: Attachment — Submitted filename: Responses to Editor and Reviewers.docx [file pone.0310898.s006.docx]

**Editor' Comments:**

i) enhance the description regarding the procedure for conducting multivariable MR;

**Oue response:** We supplemented the detailed description regarding the procedure for conducting multivariable MR. **Please see Page 6-7, *Lines 134-146.***

***(Lines 134-146):***

Importantly, multivariable MR (MVMR) was next conducted to evaluate the independent causal effects of significant anthropometric measures on sepsis outcomes. This method is an effective statistical approach, enabling us to estimate the direct effect of each phenotype on the outcome by incorporating SNPs with multiple phenotypes into the analysis(1). The variants selected for MVMR analyses were screened based the following methods: i) the least absolute shrinkage and selection operator (LASSO) regression was used to remove highly correlated exposures in the presence of various exposures, enhancing the accuracy and reliability of the results(2); ii) the variants with a Cochran’s Q p value<0.05 were removed; iii) the remaining genetic instruments for each exposure with a significant effect on outcomes in the univariable MR analyses were selected and then the MVMR analysis was then performed using “TwoSampleMR” R package. Because of the different screening criteria in the univariable and MVMR analyses, the number of IVs can be different.

ii) revise the introduction to elucidate the methodology behind the selection of genetic IVs for the multivariable MR analysis;

**Oue response:** The genetically instrumental variables (IVs) “SNPs” were screened based on the following criteria: 1) showing strong associations with exposures at a genome-wide level of significance (P<5x10^-8^); 2) without a linkage disequilibrium level (r^2^< 0.001). Afterward, selected variants were taken forward to harmonised with sepsis data and then included for multivariable MR analysis. These criteria have been supplemented in our revised manuscript. **Please see Page 6, *Lines 112-116.***

***(Lines 112-116):***

The rigorous criteria were set to screen these IVs and all SNPs that showed strong association with the anthropometric measures at genome-wide significance (P<5×10^−8^) were selected. Then we harmonized these selected variants with sepsis data, excluding palindromic SNPs and clumping for linkage disequilibrium (LD, a window size greater than 10,000 kb, r^2^<0.001)(3).

iii) integrate an overview of MR in the introduction, elucidating the three fundamental assumptions underlying MR analysis;

**Oue response:** We have supplemented a short description of MR in the introduction. Meanwhile, we also add three core assumption of MR in the method part. **Please see Page 4, *Lines 71-75;* Page 5, *Lines 105-109.***

***(Lines 71-75):*** As a key epidemiological technique, MR analysis uses genetically instrumental variables (IVs) as proxies to explore the causality of exposure on outcome of interest, which overcomes these defects and bias caused by reverse causation, confounders, and other biases in observational studies(4). Importantly, MR can distinguish the causal association that simple regression analysis is not able to reply(5).

***(Lines 105-109):*** All of MR analysis were performed according to the following three key assumptions: (a) the multiple genetic variants included significantly involve in the exposure of interest; (b) no confounders affect the relationship between the exposure and outcome; (c) the genetic variants should influence the outcome only via their effect on exposure(6).

iv) refine the introduction to minimize references to external publications and focus on elucidating the study's unique contributions, particularly concerning the investigation of body fat parameters.

***(Lines 105-109):*** We have revised our introduction and discussion according to your and reviewers’ comments. **Please see Pages 3-4, *Lines 59-70;* Page 16-17, *Lines 327-347.***

***(Lines 59-70):*** Thus, increasing studies are taking an interest in thoroughly revealing the association between specific and biological components of BMI [including fat mass, nonfat mass and height (henceforth, ‘anthropometric measures’)] and disease. For example, in order to better understand the obesity–depression association, Speed et al(7) performed a MR study of the relationship between anthropometric measures and depression, and they found that both height (short stature) and fat mass are causal risk factors for depression, while nonfat mass is not. In cardiometabolic diseases, Larsson et al(8) also demonstrated that fat mass is causally associated with the risk of major cardiovascular events, while the effect of fat-free mass on cardiometabolic diseases seems to be neutral. These findings further confirmed the importance of fat mass in diseases, meanwhile denied the role of non-fat mass. However, the association of anthropometric measures with sepsis has not been revealed.

***(Lines 327-347):***

It is worth mentioning that the association of lean mass (namely nonfat mass, mainly including muscle and bone) and body fat change with sepsis has also been revealed in recent literature. Several literatures have demonstrated that septic humans and animals are in great demand for supraphysiological energy supplies, but they are usually unwilling to eat or can’t eat. Such negative energy balance leads to the amounts of fat tissue, cellular glycogen, and muscle mass decline rapidly, in order to provide enough energy for body metabolism(9). However, these high-energy metabolites [including free fatty acids, glycerol, lactate, and gluconeogenic amino acids] can’t be transformed into useful molecules such as glucose and ketones to provide and release energy, due to the downregulation of GR and PPARα. Thus, instead of supplying energy for body, the decomposition of lean mass and fat mass leads to the accumulation and toxicity of metabolites, further causing the aggravation of disease severity and lethality in septic patients and animals. For example, Lee et al revealed that muscle mass loss/depletion was associated with increased sepsis mortality(10). Importantly, some studies further suggested that obese septic mice show a unique metabolic profile that is characterized by enhanced lipolysis when compared to lean septic mice(11). These finding indicated that there is a positive correlation of sepsis severity with body mass (including lean mass and fat mass). However, it remains unclear whether the demonstrated associations are causal. In order to reveal this mystery, we performed this MR analysis and found that genetically mediated nonfat mass and fat mass are causally associated with an increased risk of sepsis via univariable MR analyses.

**Reviewers' Comments:**

**General response:** We thank the reviewers for their time and insightful comments. We have carefully considered these comments and substantially revised the manuscript by thoroughly researching the field reviewed and clarifying the content of this manuscript to make it more comprehensive. Below, we address each of the reviewers’ questions.

**Reviewer 1**

***1.This study is a one-sample MR not because the participants are predominantly European, but because there is a sample overlap between the GWASs on the exposures and the GWAS on sepsis due to the fact that they were performed on the same cohort (UK biobank). And this poses some limitations, for example on the use of MR-Egger (see here for example:*** ***<https://doi.org/10.1093/ije/dyab084>).***

**Our response:** We appreciate the reviewer’s critical and constructive comments. It is our honour to get your help to revise our manuscript. We have read through this literature you mentioned and benefited greatly. With simulations mimicking a typical study in UK Biobank, this literature assessed the performance, in terms of bias and precision of the MR estimate, of the fixed-effect and (multiplicative) random-effects meta-analysis method, weighted median estimator, weighted mode estimator and MR-Egger regression, used in both one-sample and two sample data(12). They finally demonstrated that two-sample MR methods can be safely used for one-sample MR performed within large biobanks, expect for MR-Egger. It’s our negligence for omitting this important literature, leading to a usage error of MR-Egger. We have cited this literature and revised our analysis method in our manuscript.

***2. The procedure for multivariable MR needs to be better described, with particular attention to testing for weak instruments. You should also describe how genetic IVs were selected for the multivariable MR, for example in supplementary fig. 15 and supplementary table 3 each exposure has a different number of snps, while usually all snps associated with at least one exposure are included in the analysis before harmonization, LD based selection, weak instrument testing, etc.***

**Our response:** Thank you for your feedback and comments regarding the procedure for multivariable MR. We are very grateful that the reviewer read our article carefully. It’s our negligence for omitting these important contents. We have supplemented the specific procedure of multivariable MR in our manuscript. **Please see Page 6-7, *Lines 134-146.*** On the other hand, the genetically instrumental variables (IVs) “SNPs” were then screened based on the following criteria: 1) showing strong associations with exposures at a genome-wide level of significance (P<5x10^-8^); 2) without a linkage disequilibrium level (r^2^< 0.001). Afterward, selected variants were taken forward to harmonised with sepsis data and then included for multivariable MR analysis. These criteria have been supplemented in our revised manuscript. **Please see Page 6, *Lines 112-116.***

***(Lines 134-146):***

Importantly, multivariable MR (MVMR) was next conducted to evaluate the independent causal effects of significant anthropometric measures on sepsis outcomes. This method is an effective statistical approach, enabling us to estimate the direct effect of each phenotype on the outcome by incorporating SNPs with multiple phenotypes into the analysis(1). The variants selected for MVMR analyses were screened based the following methods: i) the least absolute shrinkage and selection operator (LASSO) regression was used to remove highly correlated exposures in the presence of various exposures, enhancing the accuracy and reliability of the results(2); ii) the variants with a Cochran’s Q p value<0.05 were removed; iii) the remaining genetic instruments for each exposure with a significant effect on outcomes in the univariable MR analyses were selected and then the MVMR analysis was then performed using “TwoSampleMR” R package. Because of the different screening criteria in the univariable and MVMR analyses, the number of IVs can be different.

***(Lines 112-116):***

The rigorous criteria were set to screen these IVs and all SNPs that showed strong association with the anthropometric measures at genome-wide significance (P<5×10^−8^) were selected. Then we harmonized these selected variants with sepsis data, excluding palindromic SNPs and clumping for linkage disequilibrium (LD, a window size greater than 10,000 kb, r^2^<0.001)(3).

***3. I would add a short description of MR, with the three assumptions, in the introduction.***

**Our response:** It’s our negligence for omitting these important contents. We have supplemented a short description of MR in the introduction. Meanwhile, we also add three core assumption of MR in the method part. Thanks very much for your careful review again. Even so, we are still really sorry for our cursoriness. **Please see Page 4, *Lines 71-75;* Page 5, *Lines 105-109.***

***(Lines 71-75):*** As a key epidemiological technique, MR analysis uses genetically instrumental variables (IVs) as proxies to explore the causality of exposure on outcome of interest, which overcomes these defects and bias caused by reverse causation, confounders, and other biases in observational studies(4). Importantly, MR can distinguish the causal association that simple regression analysis is not able to reply(5).

***(Lines 105-109):*** All of MR analysis were performed according to the following three key assumptions: (a) the multiple genetic variants included significantly involve in the exposure of interest; (b) no confounders affect the relationship between the exposure and outcome; (c) the genetic variants should influence the outcome only via their effect on exposure(6).

***4. The following sentence might be rephrased: "Here, the least absolute shrinkage and selection operator (LASSO) regression was used to remove highly correlated exposures in the presence of various exposures"***

**Our response:** We appreciate your good suggestion on improving the accuracy of the expression. We have roundly revised this sentence. **Please see Page 6-7, *Lines 134-146.***

***(Lines 134-146):***

Importantly, multivariable MR (MVMR) was next conducted to evaluate the independent causal effects of relevant anthropometric measures on sepsis outcomes. This method is an effective statistical approach, enabling us to estimate the direct effect of each phenotype on the outcome by incorporating SNPs with multiple phenotypes into the analysis(1). The variants selected for MVMR analyses were screened based the following methods: i) the least absolute shrinkage and selection operator (LASSO) regression was used to remove highly correlated exposures in the presence of various exposures, enhancing the accuracy and reliability of the results(2); ii) the variants with a Cochran’s Q p value<0.05 were removed; iii) the remaining genetic instruments for each exposure with a significant effect on outcomes in the univariable MR analyses were selected and then the MVMR analysis was then performed using “TwoSampleMR” R package. Because of the different screening criteria in the univariable and MVMR analyses, the number of IVs can be different.

***5. Line 127: "Exposures with significant adjusted p values of<0.05 were defined as exposures with potential evidence...". How did you adjust the p-values?***

**Our response:** Thanks very much for your constructive suggestions. It’s our negligence for omitting the important information. We have supplemented a short description regarding how to adjust the p-values in the methods. **Please see Page 7, *Lines 151-155.***

***(Lines 151-155):***

We performed a Bonferroni correction with 42 tests to adjust for multiple testing in MR analysis, finally yielding a significance cutoff of P≤0.001 (0.05/42). Those exposures that were statistically significant (P<0.05) before but not after correction (P≤0.001) were regarded as the suggestive correlation results.

**Reviewer #3:**

***1. There seems to be a lot of repetition in the discussion on the effects of weight and height on sepsis and many portions seem redundant. Please avoid paraphrasing discussion section of another publication (Wang et al, 2023). There is a very minimal mention of body fat parameters and specific discussion around those results.***

**Our response:** We appreciate the reviewer’s critical and constructive comments. We have deleted the repetition in the discussion on the effects of weight and height on sepsis and deleted the discussion section of another publication (Wang et al, 2023). In addition, we supplemented a discussion regarding the body fat parameters. **Please see Page 15-16, *Lines 327-347.***

***(Lines 327-347):***

It is worth mentioning that the association of lean mass (namely nonfat mass, mainly including muscle and bone) and body fat change with sepsis has also been revealed in recent literature. Several literatures have demonstrated that septic humans and animals are in great demand for supraphysiological energy supplies, but they are usually unwilling to eat or can’t eat. Such negative energy balance leads to the amounts of fat tissue, cellular glycogen, and muscle mass decline rapidly, in order to provide enough energy for body metabolism(9). However, these high-energy metabolites [including free fatty acids, glycerol, lactate, and gluconeogenic amino acids] can’t be transformed into useful molecules such as glucose and ketones to provide and release energy, due to the downregulation of GR and PPARα. Thus, instead of supplying energy for body, the decomposition of lean mass and fat mass leads to the accumulation and toxicity of metabolites, further causing the aggravation of disease severity and lethality in septic patients and animals. For example, Lee et al revealed that muscle mass loss/depletion was associated with increased sepsis mortality(10). Importantly, some studies further suggested that obese septic mice show a unique metabolic profile that is characterized by enhanced lipolysis when compared to lean septic mice(11). These finding indicated that there is a positive correlation of sepsis severity with body mass (including lean mass and fat mass). However, it remains unclear whether the demonstrated associations are causal. In order to reveal this mystery, we performed this MR analysis and found that genetically mediated nonfat mass and fat mass are causally associated with an increased risk of sepsis via univariable MR analyses.

***2. - The sample overlap bias alluded to in the previous review has not been sufficiently addressed in the authors' responses especially if the authors state that this is a one sample MR. Why is two sample MR package being mentioned in the statistical section, it is a bit confusing. I think authors need to address this in a revision.***

**Our response:** Thanks very much for your critical question. You are right, this study is a one-sample MR due to the fact that they were performed on the same cohort (UK biobank). However, the feasibility of two sample MR package being used in one-sample MR analysis has been demonstrated by Pro. Minelli and coworkers(12). With simulations mimicking a typical study in UK Biobank, they assessed the performance, in terms of bias and precision of the MR estimate, of the fixed-effect and (multiplicative) random-effects meta-analysis method, weighted median estimator, weighted mode estimator and MR-Egger regression, used in both one-sample and two sample data. They finally demonstrated that two-sample MR methods can be safely used for one-sample MR performed within large biobanks, expect for MR-Egger. We have cited this literature and revised our analysis method in our manuscript.

***3. While the results and analysis section is satisfactory, introduction and discussion needs to improve.***

**Our response:** Thanks for your recognition. We have revised our introduction and discussion according to you and other reviewers’ comments. Again, appreciate your work and review. Your comments give us valuable assistance in improving our manuscript. We have carefully revised our manuscript accordingly. After adopting your suggestions, we believe a significant improvement can be seen in our revised manuscript. Best wishes! **Please see Pages 3-4, *Lines 59-70;* Page 16-17, *Lines 327-347.***

***(Lines 59-70):*** Thus, increasing studies are taking an interest in thoroughly revealing the association between specific and biological components of BMI [including fat mass, nonfat mass and height (henceforth, ‘anthropometric measures’)] and disease. For example, in order to better understand the obesity–depression association, Speed et al(7) performed a MR study of the relationship between anthropometric measures and depression, and they found that both height (short stature) and fat mass are causal risk factors for depression, while nonfat mass is not. In cardiometabolic diseases, Larsson et al(8) also demonstrated that fat mass is causally associated with the risk of major cardiovascular events, while the effect of fat-free mass on cardiometabolic diseases seems to be neutral. These findings further confirmed the importance of fat mass in diseases, meanwhile denied the role of non-fat mass. However, the association of anthropometric measures with sepsis has not been revealed.

***(Lines 327-347):***

It is worth mentioning that the association of lean mass (namely nonfat mass, mainly including muscle and bone) and body fat change with sepsis has also been revealed in recent literature. Several literatures have demonstrated that septic humans and animals are in great demand for supraphysiological energy supplies, but they are usually unwilling to eat or can’t eat. Such negative energy balance leads to the amounts of fat tissue, cellular glycogen, and muscle mass decline rapidly, in order to provide enough energy for body metabolism(9). However, these high-energy metabolites [including free fatty acids, glycerol, lactate, and gluconeogenic amino acids] can’t be transformed into useful molecules such as glucose and ketones to provide and release energy, due to the downregulation of GR and PPARα. Thus, instead of supplying energy for body, the decomposition of lean mass and fat mass leads to the accumulation and toxicity of metabolites, further causing the aggravation of disease severity and lethality in septic patients and animals. For example, Lee et al revealed that muscle mass loss/depletion was associated with increased sepsis mortality(10). Importantly, some studies further suggested that obese septic mice show a unique metabolic profile that is characterized by enhanced lipolysis when compared to lean septic mice(11). These finding indicated that there is a positive correlation of sepsis severity with body mass (including lean mass and fat mass). However, it remains unclear whether the demonstrated associations are causal. In order to reveal this mystery, we performed this MR analysis and found that genetically mediated nonfat mass and fat mass are causally associated with an increased risk of sepsis via univariable MR analyses.

**Reference**

1.	Sanderson E, Davey Smith G, Windmeijer F, Bowden J. An examination of multivariable Mendelian randomization in the single-sample and two-sample summary data settings. International journal of epidemiology. 2019;48(3):713-27.

2.	Sun Z, Tao Y, Li S, Ferguson KK, Meeker JD, Park SK, et al. Statistical strategies for constructing health risk models with multiple pollutants and their interactions: possible choices and comparisons. Environmental health : a global access science source. 2013;12(1):85.

3.	J B, J T, R G. The Rough Guide to Genes & Cloning. 2007.

4.	Haycock PC, Burgess S, Wade KH, Bowden J, Relton C, Davey Smith G. Best (but oft-forgotten) practices: the design, analysis, and interpretation of Mendelian randomization studies. The American journal of clinical nutrition. 2016;103(4):965-78.

5.	Davey Smith G, Hemani G. Mendelian randomization: genetic anchors for causal inference in epidemiological studies. Human molecular genetics. 2014;23(R1):R89-98.

6.	Aad G, Abbott B, Abdallah J, Abdinov O, Aben R, Abolins M, et al. Search for Dark Matter in Events with Missing Transverse Momentum and a Higgs Boson Decaying to Two Photons in pp Collisions at sqrt[s]=8 TeV with the ATLAS Detector. Physical review letters. 2015;115(13):131801.

7.	Speed MS, Jefsen OH, Børglum AD, Speed D, Østergaard SD. Investigating the association between body fat and depression via Mendelian randomization. Translational psychiatry. 2019;9(1):184.

8.	Larsson SC, Burgess S. Fat mass and fat-free mass in relation to cardiometabolic diseases: a two-sample Mendelian randomization study. Journal of internal medicine. 2020;288(2):260-2.

9.	Rosenthal MD, Moore FA. Persistent inflammatory, immunosuppressed, catabolic syndrome (PICS): A new phenotype of multiple organ failure. Journal of advanced nutritional and human metabolism. 2015;1(1).

10.	Lee Y, Park HK, Kim WY, Kim MC, Jung W, Ko BS. Muscle Mass Depletion Associated with Poor Outcome of Sepsis in the Emergency Department. Annals of nutrition & metabolism. 2018;72(4):336-44.

11.	Goossens C, Weckx R, Derde S, Dufour T, Vander Perre S, Pauwels L, et al. Adipose tissue protects against sepsis-induced muscle weakness in mice: from lipolysis to ketones. Critical care (London, England). 2019;23(1):236.

12.	Minelli C, Del Greco MF, van der Plaat DA, Bowden J, Sheehan NA, Thompson J. The use of two-sample methods for Mendelian randomization analyses on single large datasets. International journal of epidemiology. 2021;50(5):1651-9.
