# Supplementary material for: Mendelian randomization analysis reveals causal association of anthropometric measures on sepsis risk and mortality
Source: PLoS One. 2024 Sep 30;19(9):e0310898. doi: 10.1371/journal.pone.0310898 (PMC11441680; doi:10.1371/journal.pone.0310898)
Supplement: Attachment — Submitted filename: Responses to Editor and Reviewers.docx [file pone.0310898.s007.docx]

**Editor' Comments:**

**The authors have addressed most of the concerns raised by the reviewer. However, there are few minor comments that need to be addressed.**

**Specifically:**

**i) the issue with sample overlap is not addressed in the manuscript;**

**ii) the reference cited in the response to reviewers was not added to the manuscript;**

**iii) it should be included that MR is not immune to reverse causation;**

**iv) genetic associations should be included in supplementary table 1;**

**v) the conclusion of the study needs better explanation.**

**Our response:** Thank you for taking the time to review our article. We have provided point-by-point responses to each of the reviewers’ questions. Please find them below. Thanks very much again! If there are any more problems, please do not hesitate to contact us. Best wishes!

**Reviewers' Comments:**

**General response:** We thank the reviewers for their time and insightful comments. We have carefully considered these comments and substantially revised the manuscript by thoroughly researching the field reviewed and clarifying the content of this manuscript to make it more comprehensive. Below, we address each of the reviewers’ questions.

**Reviewer #1: *Thank you for the revisions made and for incorporating additional details on data analysis, such as sensitivity analysis. This manuscript effectively demonstrates a causal relationship between body mass and sepsis, providing valuable insights that could inform future clinical management strategies.***

**Our response:** We appreciate the reviewer’s critical and constructive comments. It is our honour to get your help to revise our manuscript. Thanks very much for your recognition. Best wishes!!

***Reviewer #2:***

***1.The sample overlap issue was not addressed in the manuscript, and the reference you cite in the response to reviewers was not added to the manuscript.***

**Our response:** Thank you for your feedback and comments regarding the sample overlap issue. We are very grateful that the reviewer read our article carefully. As pointed previously, Pro. Minelli and coworkers(1) has demonstrated the feasibility of two sample MR package being used in one-sample MR analysis in 2021, thus after that some MR studies using the same cohort (such as UK biobank) were accepted for publication(2, 3). But it’s our negligence for omitting the important literature in our manuscript. We have added it in our revised manuscript. **Please see Page 4, *Lines 78-81.***

***(Lines 78-81):***

Importantly, Pro. Minelli and coworkers (1) further demonstrated the feasibility of two sample MR package being used in one-sample MR analysis in 2021, which promoting the application and development of MR in medical field(2, 3).

***2. Introduction, line 71. I suggest to move the MR assumptions here.***

**Our response:** Thanks for your great suggestion, and we have moved the MR assumptions according to your advice. **Please see Page 4, *Lines 71-75.***

***(Lines 71-75):***

All of MR analysis were performed according to the following three key assumptions: (a) the multiple genetic variants included significantly involve in the exposure of interest; (b) no confounders affect the relationship between the exposure and outcome; (c) the genetic variants should influence the outcome only via their effect on exposure(4).

***3. Introduction, line 73. MR is not immune the reverse causation, which can be tested by bidirectional MR.***

**Our response:** We appreciate your good suggestion on improving the accuracy of the expression. We have deleted this incorrect expression. **Please see Page 4, *Lines 75-78.***

***(Lines 75-78):***

As a key epidemiological technique, MR analysis uses genetically instrumental variables (IVs) as proxies to explore the causality of exposure on outcome of interest, which overcomes these defects and bias caused by confounders, and other biases in observational studies(5).

***4. Introduction, line 75. With the right study design, you can test for causality with a regression (e.g., randomized controlled trials, lab experiment).***

**Our response:** Thanks very much for your constructive instruction. We have deleted this sentence. Your professional comments give us inspiration. Thanks very much!

***5. Methods, line 92: supplementary table 1 does not contain genetic associations***

**Our response:** We appreciate the reviewer’s constructive comments. We have supplemented the genetic associations (including number of SNP, approximate variance explained, and mean F-statistic) in our supplementary table 1. **Please see the following table:**

***6. Methods, line 105: see comment above***

**Our response:** We have revised this according to your instruction above.

***7. Discussion: you start the discussione by saying (line 276-277): "...Our findings indicated that weight-related measures are closely related to sepsis risk and mortality..." and end it with "..but MVMR analysis indicated the observed effects for weight-related measures in the univariable MR analyses are more likely a bias caused by the interrelationship between anthropometric measures...". It is not easy to understand the conclusions that the authors are drawing.***

**Our response:** We appreciate the reviewer’s critical comments. Just as pointed in our discussion, a new term “obesity paradox” is now popular in academic world. The reason is that high BMI is demonstrated to contribute to worse outcomes for critically ill patients in early works(6, 7), while a relationship with improved outcomes with an raised BMI was found in some more recent work(8-10). The underlying mechanism of this phenomenon remains unclear and understanding the genetic role of sepsis is perhaps the key to unravel the mysterious relationship between obesity and outcomes in patients with sepsis. Our results from univariable MR analysis supported the positive association of BMI with sepsis mortality at 28 days, which was in accordance with the outcomes from other literatures(11). However, these MR analyses failed to control for pleiotropic pathways resulted from the multicollinearity among anthropometric measures. Thus, we further performed MVMR analyses to assess the independent causal effects of BMI on sepsis risk utilizing lasso regression method. Our results demonstrated that the direct estimates for the BMI were significantly attenuated, causing a wider 95% CI that overlapped null. Hence, the observed effects for BMI in the univariable MR analyses are more likely a bias caused by the interrelationship between anthropometric measures. **This probably explain the reason why “obesity paradox” occur in sepsis: namely previous literatures only paid attention to the association between BMI and sepsis and did not consider the collinear effect from other anthropometric measures.** Thus, we concluded that these findings represent vital new knowledge on the role of anthropometric-related measures in the sepsis etiology.

**Reference**

1.	Minelli C, Del Greco MF, van der Plaat DA, Bowden J, Sheehan NA, Thompson J. The use of two-sample methods for Mendelian randomization analyses on single large datasets. International journal of epidemiology. 2021;50(5):1651-9.

2.	Lei P, Xu W, Wang C, Lin G, Yu S, Guo Y. Mendelian Randomization Analysis Reveals Causal Associations of Polyunsaturated Fatty Acids with Sepsis and Mortality Risk. Infectious diseases and therapy. 2023;12(7):1797-808.

3.	Almramhi MM, Storm CS, Kia DA, Coneys R, Chhatwal BK, Wood NW. The role of body fat in multiple sclerosis susceptibility and severity: A Mendelian randomisation study. Multiple sclerosis (Houndmills, Basingstoke, England). 2022;28(11):1673-84.

4.	Aad G, Abbott B, Abdallah J, Abdinov O, Aben R, Abolins M, et al. Search for Dark Matter in Events with Missing Transverse Momentum and a Higgs Boson Decaying to Two Photons in pp Collisions at sqrt[s]=8 TeV with the ATLAS Detector. Physical review letters. 2015;115(13):131801.

5.	Haycock PC, Burgess S, Wade KH, Bowden J, Relton C, Davey Smith G. Best (but oft-forgotten) practices: the design, analysis, and interpretation of Mendelian randomization studies. The American journal of clinical nutrition. 2016;103(4):965-78.

6.	Rattan R, Nasraway SA, Jr. Separating wheat from chaff: examining the obesity paradox in the critically ill. Critical care (London, England). 2013;17(4):168.

7.	Nasraway SA, Jr., Albert M, Donnelly AM, Ruthazer R, Shikora SA, Saltzman E. Morbid obesity is an independent determinant of death among surgical critically ill patients. Crit Care Med. 2006;34(4):964-70; quiz 71.

8.	Sakr Y, Alhussami I, Nanchal R, Wunderink RG, Pellis T, Wittebole X, et al. Being Overweight Is Associated With Greater Survival in ICU Patients: Results From the Intensive Care Over Nations Audit. Crit Care Med. 2015;43(12):2623-32.

9.	Christopher KB. The Body Mass Index Paradox. Crit Care Med. 2015;43(12):2693-4.

10.	Yeo HJ, Kim TH, Jang JH, Jeon K, Oh DK, Park MH, et al. Obesity Paradox and Functional Outcomes in Sepsis: A Multicenter Prospective Study. Crit Care Med. 2023;51(6):742-52.

11.	Butler-Laporte G, Harroud A, Forgetta V, Richards JB. Elevated body mass index is associated with an increased risk of infectious disease admissions and mortality: a mendelian randomization study. Clinical microbiology and infection : the official publication of the European Society of Clinical Microbiology and Infectious Diseases. 2020.
